# BAR Proteins PSTPIP1/2 Regulate Podosome Dynamics and the Resorption Activity of Osteoclasts

**DOI:** 10.1371/journal.pone.0164829

**Published:** 2016-10-19

**Authors:** Martin Sztacho, Sandra Segeletz, Maria Arantzazu Sanchez-Fernandez, Cornelia Czupalla, Christian Niehage, Bernard Hoflack

**Affiliations:** Biotechnology Center, Technische Universität Dresden, Tatzberg 47–51, 01307, Dresden, Germany; Universite de Nantes, FRANCE

## Abstract

Bone resorption in vertebrates relies on the ability of osteoclasts to assemble F-actin-rich podosomes that condense into podosomal belts, forming sealing zones. Sealing zones segregate bone-facing ruffled membranes from other membrane domains, and disassemble when osteoclasts migrate to new areas. How podosome/sealing zone dynamics is regulated remains unknown. We illustrate the essential role of the membrane scaffolding F-BAR-Proline-Serine-Threonine Phosphatase Interacting Proteins (PSTPIP) 1 and 2 in this process. Whereas PSTPIP2 regulates podosome assembly, PSTPIP1 regulates their disassembly. PSTPIP1 recruits, through its F-BAR domain, the protein tyrosine phosphatase non-receptor type 6 (PTPN6) that de-phosphophorylates the phosphatidylinositol 5-phosphatases SHIP1/2 bound to the SH3 domain of PSTPIP1. Depletion of any component of this complex prevents sealing zone disassembly and increases osteoclast activity. Thus, our results illustrate the importance of BAR domain proteins in podosome structure and dynamics, and identify a new PSTPIP1/PTPN6/SHIP1/2-dependent negative feedback mechanism that counterbalances Src and PI(3,4,5)P3 signalling to control osteoclast cell polarity and activity during bone resorption.

## Introduction

Bone remodeling is a key process that occurs continuously throughout life, needed during the development, maintenance and repair of the skeleton of vertebrates. It involves the coordinated activity of bone-building osteoblasts and bone-digesting osteoclasts. An unbalanced interaction between these two cell types results in disabling diseases such as osteopetrosis, osteopenia or osteoporosis. Osteoclasts are multinucleated cells arising from hematopoietic, mono-nucleated precursors. Macrophage-stimulating factor (M-CSF) triggers the proliferation of these precursors, and the cytokine receptor-activator of NF-κB ligand (RANKL) induces their differentiation into cells able to fuse with each other to generate multi-nucleated osteoclasts [1]. To digest large bone surface areas, mature osteoclasts create between their bone-facing ruffled membrane and the bone surface an acidic resorption lacuna, into which lysosomal hydrolases are delivered. The formation of resorption lacunae relies on podosomes, F-actin-rich structures linking cell adhesion molecules and actin meshworks. Multiple podosomal units condense into compact podosomal belts, which form sealing zones that segregate the ruffled membrane from other membrane domains [2]. These podosomal belts and sealing zones disassemble when osteoclasts migrate to digest other bone areas. Thus, cycles of bone digestion and cell migration are linked to the dynamic assembly and disassembly of these F-actin-rich structures [3].

Podosomes have been detected in several cell types including osteoclasts. They share many components with the focal adhesions of adhesive cells, or with invadopodia, that cancer cells assemble in order to digest the extracellular matrix during invasion and metastasis [4–6]. How podosomes, focal adhesions and invadopodia are similar in their structural organization is not clear. However, it has been firmly established that podosome and sealing zone assembly in osteoclasts depends on Src-dependent phosphorylation. Src-/- mice develop osteopetrosis due to the inability of osteoclasts to form podosomes and sealing zones [7]. Using quantitative mass spectrometry-based proteomics, we have previously identified Src substrates in osteoclasts, including the Proline-Serine-Threonine Phosphatase Interacting Proteins 1 and 2 (PSTPIP1/2) [8]. PSTPIP1/2 are mostly expressed in the myeloid lineage [9]. They exhibit ≈60% amino acid sequence identity and contain putative F-BAR domains that sense membrane curvature [10, 11]. However, the structure of these two isoforms differs due to the presence of a SH3 domain at the C-terminus of PSTPIP1. Mutations in the *PSTPIP1* gene cause the Pyogenic Arthritis with Pyoderma gangrenosum and Acne (PAPA) syndrome, a dominantly inherited human auto-inflammatory disorder characterized by a destructive inflammation of the skin and joints, due to defects in macrophage migration [12]. Mutations in PSTPIP2 are associated with the autoinflammatory disorder chronic multifocal osteomyelitis in mice [13]. PSTPIP2 has been proposed to be a negative regulator of Tartrate-resistant acid phosphatase expression and osteoclast precursor fusion [9]. We now illustrate the functional importance of PSTPIP1/2 in podosome/sealing zone dynamics and osteoclast activity. Using quantitative mass spectrometry-based proteomics, we identified some of their interacting partners. We illustrate the function of the PSTPIP1/PTPN6/SHIP1/2 complex. We confirm our findings by conditionally knockingout PSTPIP1 in mouse osteoclasts.

## Material and Methods

### Reagents

Primary antibodies: mouse monoclonal antibodies against phosphotyrosine (clone 4G10 Millipore, Temecula, CA; 1:1000 western blotting; 1:500 immunofluorescence), PSTPIP1 (clone 1D5, Abnova, Taipei, Taiwan; 1:500 western blotting), SHIP1 (Santa Cruz, Santa-Cruz, USA; 1:300 western blotting; 1:200 immunofluorescence), GAPDH (Acris Antibodies, Herford, Germany; 1:500 western blotting), phosphatidylinositol 3,4,5-diphosphate (clone RC6F8, Eugene, USA; 1:300 immunofluorescence); mouse polyclonal antibody against PTPN18 (Abnova Corp., Taipei, Taiwan, 1:100 immunofluorescence); rabbit polyclonal antibodies against PTPN12 (Abcam plc, Cambridge, UK; 1:500 western blotting; 1:300 immunofluorescence), PSTPIP2 (Atlas Antibodies, Sigma-Aldrich, St. Louis, USA 1:400 western blotting; 1:300 immunofluorescence), PTPN22 (Santa Cruz, Santa-Cruz, USA; 1:100 immunofluorescence), PTPN6 (Atlas Antibodies, Sigma-Aldrich, St. Louis, USA; 1:300 western blotting, 1:200 immunofluorescence), SHIP2 (Atlas Antibodies, Stockholm, Sweden; 1:300 western blotting, 1:200 immunofluorescence); goat polyclonal PSTPIP1 (Santa Cruz, Santa-Cruz, USA; 1:100 immunofluorescence),

Secondary antibodies: Horse-radish peroxidase (HRP) -conjugated goat anti-mouse IgG (1:5000 western blotting), goat anti-rabbit IgG (1:5000) (Jackson Immuno Research Laboratories, Suffolk, UK), Alexa Fluor 488, 546, 633-conjugated against the corresponding primary antibodies (1:400) (Molecular Probes, Invitrogen, Darmstadt, Germany).

### Constructs

GFP-tagged PSTPIP1, PSTPIP2 constructs, RFP-tagged Actin constructs were amplified by PCR and subcloned into the pGFP, pRFP vector (Clonetech). GST-tagged F-BAR domain of PSTPIP1 (aa 1-262), SH3 domain of PSTPIP1 (aa 351-416), full-length GST-PSTPIP1 and GST-PSTPIP2 were subcloned into the pGEX-4T-1 vector (GE Healthcare).

### Cell Culture

HEK293A (Qbiogene), Raw264.7 (American Type Culture Collection (ATCC), Wesel, Germany), were cultivated at 37°C in humidified 5% CO_2_ atmosphere in Dulbecco’s modified Eagle medium (DMEM)-GlutaMAX-I (Invitrogen, Darmstadt, Germany). Primary mouse bone marrow cells were cultivated at 37°C in humidified 10% CO_2_ atmosphere in alphaMEM media. Media were supplemented with 10% (v/v) Superior Fetale Bovine Serum (Biochrom), 2 mM L-glutamine, 0.1 mg/ml streptomycin and 100 U/ml penicillin-G (Invitrogen, Darmstadt, Germany).

### RNA Interference and Gene Transduction

In vitro osteoclastogenesis was induced by addition of RANKL (3% (v/v) ~ 50ng/ml) to Raw264.7 cells, plated at a density of 1x10^6^ cells. At day four, cells were differentiated into osteoclasts. For microscope analysis, differentiated cells were washed 2 times with PBS and then incubated for 15min with PBS to remove undifferentiated mononucleated cells. Then osteoclasts were detached with a cell lifter (Corning Incorporated Costar). Detached cells were centrifuged at 220 g for 5 min, resuspended in DMEM/3% RANKL, and then transferred either to glass cover slips (∅ 11mm) or to BD BioCoat osteological^™^ discs (BD Bioscience), Ibidi μm, 35mm, glass bottom dish, hydroxyapatite coated chambers or subjected to electoporation. Soluble recombinant RANKL was produced in *Pichia* yeast as described previously (Czupalla et al., 2005).

HEK293a cells were seeded on glass cover slips (∅ 11 mm, Menzel) 24h before transfection. HeLa cells seeded at a density 3x10^4^ cells/mL were transfected with a mixture of 1μg plasmid DNA and 3μL cationic polyethylenimine transfection reagent JetPEI^™^ (Peqlab Biotechnologie GmbH). 24h post transfection cells were processed for subsequent analysis.

Stealth RNAi duplexes were designed according to Invitrogen’s BLOCK-iT algorithm and purchased from Invitrogen. After 4 days of differentiation on plastic dishes. Osteoclasts were detached as described, centrifuged for 5min at 220g, and resuspended in Electroporation Isoosmolar Buffer (Eppendorf). 1μM of predesigned stealth RNAi or Negative Control Medium GC stealth RNAi duplexes (Invitrogen) were electroporated into osteoclasts with a single square wave pulse of 2750 V/cm field strength and 0.4ms pulse length using an ECM830 ElectroSquarePorator^™^ (BTX, Harvard Apparatus). Electroporated osteoclasts were resuspended in DMEM/3% RANKL and allowed to recover for 46hrs. Osteoclasts were processed for subsequent analysis. At least three stealth RNA I duplexes were tested to silence any given gene. Below are listed the stealth RNAi duplexes used in this study:

**PSTPIP1#1 (NM_011193.2):** position 640: 5’- gaugaauucccugaggaccuccuuu -3’

**PSTPIP1#2 (NM_011193.2):** position 759: 5’- gagagagacagaaagagacagcggaa -3’

**PSTPIP2#1 (NM_013831.4):** position 230: 5’- uucaggugcuggauaaugcugucgu -3’

**PTPN6#1 (NM_013545.2):** position 1016: 5’- gcuacaagaacauucuucccuuuga -3’

**PTPN12#1 (NM_011203.2):** position 549: 5’- ugaaguagucgguucuugcuuguuc -3’

**PTPN22#1 (NM_008979):** position 169: 5’- uucacuggcaaacuccucacuguug -3’

**SHIP1#1 (NM_51742.1):** position 901: 5’- gacaagucugcugucuuccauugaa -3’

**SHIP1#2 (NM_51742.1):** position 579: 5’- uguagacgcugaaacagugucucgg -3’

**SHIP2#1 (NM_162781.1.1):** position 1214: 5’- uuuagggccuucuucuggaugccug -3’

**SHIP2#2 (NM_162781.1.1):** position 1686: 5’- caugggaaguguaccaccacccaaa -3’

### Lipofection of Osteoclasts

Raw264.7 cells were seeded on Ibidi μm, 35mm glass bottom dish in RANKL containing media, at density 20x10^4^ cells/ml. After 48 h transfection by Lipofectamin 2000 (Invitrogen) was performed according to manufacturers protocol. 48 h post transfection osteoclasts were analysed by TIRF microscopy.

#### Adenovirus production and gene transduction into osteoclasts

Raw264.7 cells were seeded on Ibidi μm, 35 mm glass bottom dish at density 20x10^4^ cells/ml or after 4 days of differentiation seeded onto BD BioCoat osteological^™^ discs as described, in RANKL containing media. After 48 h, transfection by Lipofectamin 2000 (Invitrogen) or Transficient^™^ DNA Transfection Reagent (MBL) was performed according to manufacturer’s protocol. 48 h post transfection osteoclasts were analysed by TIRF microscopy or confocal microscopy.

Adenoviral vectors and recombinant adenoviruses were generated using the AdEasy^™^ system (QBIOgene) developed by He et al. [14]. Target genes were subcloned from various vectors into the transfer vector pShuttle-CMV. For homologous recombination with the pAdEasy-1 plasmid that encodes the Adenovirus-5 genome (E1/E3 deleted), pShuttle-CMV was linearised with PmeI (New England BioLabs^®^) and 100ng of linearised DNA was electroporated into 33μL of electrocompetent BJ5183-AD1 cells using 2.5 kV, 2mm cuvettes. The recombinant adenoviral construct was then cleaved with PacI and 3μg were transfected into 0.6x10^6^ HEK293A cells by using JetPEI^™^ for production of virus particles. The first virus particles were collected after 10-15 days post transfection. At least two steps of virus amplification were necessary to achieve a good virus titer. Sufficient first viral amplification was harvested from 175 cm^2^ of culture, whereas, at least ten 175 cm^2^ flasks of HEK293A cells were required for infection with the recombinant adenovirus construct in the second viral amplification and harvested after 2-5 days. Viral particles were released from the cells by 3 quick freeze/thaw/cycles using liquid nitrogen. Viral particles were purified and concentrated using a discontinuous iodixanol gradient (OptiPrep^®^, Axis Shield) adapting the method from Zolotukhin et al (Zolotukhin et al., 1999). The purified virus solution was supplied with 1/3, 3x storage buffer (15mM Tris pH8.0, 150mM NaCl, 0.15% BSA, 50% (v/v) glycerol), and 1/3 of glycerol. The viruses were stored at –20°C. After 4 days of differentiation osteoclasts were transduced with titrated adenovirus and grown for additional 48 h either plated on BD BioCoat osteological^™^ discs. Then cells were processed for subsequent analysis.

### Immunocytochemistry

For immunocytochemistry analysis, cells were grown on glass cover slips (∅ 11 mm) or BioCoat osteological^™^ discs and fixed with 3% (w/v) paraformaldehyde in PBS for 15min at 37°C, quenched with 50mM NH_4_Cl/PBS for 10 minutes and permeabilised with 0.1% (w/v) Triton X-100 in PBS for 6min. Samples were blocked with 3% (w/v) BSA in PBS for 30 min at room temperature. Then, cover slips were transferred into a humid chamber and incubated with 30μl primary antibody diluted in 3% (w/v) BSA in PBS for 1h at room temperature. Following three PBS washing steps, cover slips were incubated, in the dark, with the appropriate secondary antibody as described above. Staining of the actin cytoskeleton was performed with Phalloidin Alexa 546 or Phalloidin Alexa 633 (Invitrogen). Coverslips were washed five times with PBS, once with water and mounted on glass slides by inverting them onto a droplet of Mowiol containing 10μg/mL DAPI (Invitrogen). BioCoat osteological^™^ discs were washed five times with PBS and once with water and mounted as following: 1droplet of Mowiol was added to the glass slide, then the BioCoat osteological^™^ discs were placed with the cells facing the top, 20μL Mowiol+DAPI and a glass slide with nail polish droplets was inverted on the top of the BioCoat osteological^™^ discs.

### Immunocytochemistry with PIP_3_ IgM

Cells were fixed by 4% PFA for 30 min at 37°C. Permeabilization and blocking were performed simultaneously by in 0.5% saponine in 3% BSA in PBS on ice for 45 min. From this point work was done on ice. Primary IgM PIP3 antibody (RC6F8) in 0.5% saponine in 3% BSA in PBS buffer was added onto fixed cells and stained for 1 hr (Chen et al., 2002). Cells were washed 3x by ice cold PBS and appropriated secondary antibody was added. After 30 min cells were 4x washed by ice cold PBS. Post fixation was done by 10 min incubation with 2% PFA on ice.

### Confocal Laser Scanning Microscopy and Fluorescence Microscopy

Imaging was performed on an inverted confocal laser scanning microscope Zeiss LSM 780, upright with plan-Apochromat 40x/ 1,4 oil, plan-Apochromat 63x/1,46 oil, and plan-Apochromat 100x/ 1,46 oil objectives. Zeiss 510 confocal microscope with HCX PL APO 40x/1.25-0.75 Oil, HCX PL APO 63x/1.4-0.6 Oil, HCX PL APO 100x/1.4 Oil objectives. Zeiss Apotome inverse with LD plan Neofluar 20x/ 0,4 dry, LD plan Neofluar 40x/ 0,6 dry, and EC plan Neofluar 40x/ 1,3 oil objectives. Leica AFLX6000 TIRF with HCX PL APO 100x/ 1,47 oil. 4D live cell imaging system with Plan Apochromat 100x/ 1,4 Oil, EC Plan Neofluar 40x/ 1,3 Oil, and Plan Apochromat 63x/ 1,4 Oil objectives. Images were generally taken as 8Bit 1024/1024 image frames. To prevent cross-contamination between fluorochromes, each channel was imaged sequentially using the multitrack recording module before merging.

### Time-Lapse Videomicroscopy

Raw 264.7 cells derived osteoclasts were transferred to coverslips coated with hydroxyapatite (BD Biosciences, Heidelberg, Germany) and grown in Minimum Essential Eagle Medium (Sigma-Aldrich, St. Louis, USA) supplemented with 10% FCS and 3% soluble recombinant RANKL. Cells were transfected by adenovirus coding RFP- tagged actin binding domain of Ezrin protein and observed with a Zeiss Axiovert 200 M inverted microscope equipped with an automated stage, an Incubator XL3 for temperature maintenance and CO_2_ buffering (PeCon). Sequential images were acquired every 1 min for 30 min and processed with the MetaMorph version 6.1 Imaging software (Molecular Devices, Sunnyvale, USA). Sealing zone diameters were assessed using Fiji software and statistical significance was tested using unpaired students t-test (Graph Pad Prism 6).

For monitoring of individual podosomes Raw 264.7 cells were seeded on Ibidi μm, 35mm glass bottom dish in RANKL containing media, at density 20x104 cells/ml. After 48 h transfection by Lipofectamin 2000 (Invitrogen) was performed according to manufacturer’s protocol. 48 h post transfection osteoclasts were analysed by Leica AFLX6000 TIRF. Sequential images were acquired every 2 sec for 5 min.

### Image Analysis

Images from fluorescence and confocal acquisitions were processed with Adobe Photoshop v7.0 (Adobe Systems). All image processing and analysis were carried out with FIJI software (Schindelin et al., 2012)

### Immunoprecipitation

Cells were washed with PBS, harvested in ice-cold lysis buffer (50 mM Tris pH 7.5, 150 mM NaCl, 1% (v/v) NP-40, 0.1% (w/v) sodium deoxycholate, 1 mM EDTA, 10 mM sodium β-glycerophosphate, 10 mM NaF, 1 mM sodium orthovanadate, and protease inhibitors (CompleteTM tablets, Roche Diagnostics, Mannheim, Germany), homogenized by resuspension first three times with a 22.5 Gauge needle, second five times with a 27 Gauge needle, then lysed for 15 min on a rotating wheel at 4°C, and centrifuged at 14,000 g at 4°C for 10 min. Protein concentrations of the lysate supernatants were estimated using the DC protein assay including reagents S, A, and B (Bio-Rad, Munich, Germany).

For immunoprecipitation with specific antibodies (PSTPIP1, PSTPIP2), lysates (total protein concentration > 2 mg/ml) were precleared on Protein G-sepharose beads for 1 h at 4°C. Supernatants were incubated with antibodies (5 μg/mg of lysate) for 1 h at 4°C. Then, Protein G-sepharose beads were added for additional 2 h at 4°C. Precipitates were washed two times with lysis buffer, proteins were eluted, and resolved on SDS- PAGE. For immunoblotting, gel-separated proteins were transferred onto nitrocellulose membranes and incubated with the corresponding antibodies. After incubation with secondary antibodies conjugated with HRP (Jackson Immuno Research, Suffolk, UK), bands were detected with enhanced chemiluminescence (ECL) Western Blotting Detection Reagents (GE healthcare, München, Germany).

For immunoprecipitation of tyrosine phosphorylated proteins followed by SILAC mass spectrometry-based analysis (see below), lysates of treated and non-treated cells were combined in 1:1 ratio, for immunoprecipitation of tyrosine phosphorylated proteins followed by immunoblotting, lysates were kept separated. Such lysates (total protein concentration > 2 mg/ml) were precleared on Protein A-sepharose beads for 2 h at 4°C. Supernatants were incubated with immobilized anti-phosphotyrosine antibodies (25 μg 4G10/mg of lysate and 10 μl P- Tyr-100/mg of lysate) for 6 h at 4°C. Precipitates were washed four times with lysis buffer, proteins were eluted, and resolved on SDS-PAGE. For immunoblotting, gel- separated proteins were transferred onto nitrocellulose membranes and incubated with the corresponding antibodies.

### GST-Tagged Protein Purification

To 250 ml of LB overnight culture were added 750 ml of fresh media with appropriate antibiotics and shake at 190 rpm for 3,5 hrs at 37°C. Protein expression was induce with 0,1 mM IPTG and grown overnight at 15°C. Bacteria were sedimented by centrifugation 4000g for 20 min at 4°C. Pellet was washed with 1x volume LB2 BFR (5 mM Tris, 15 mM NaCl pH 8) and resuspended in LB1 BFR1 (50 mM Tris, 150 NaCl, pH 8, complete protease inhibitors, 5 u Benzonase). Suspension was lysed on French press for 5 min at 10000 kPa. Lysate was centrifuged for 30 min, 10 000g at 4°C. Sepharose beads were washed 2x with 500 ul with water and 2x with LB1 BFR. 50% beads slurry was prepared by adding 250 ul of LB1 BFR to washed beads. Supernatant from bacterial lysate was filtered through 0,45 um and added to washed 50% bead slurry and rotated for 2 hrs at 4°C. After this incubation beads were washed 3x 500 ul with LB1 BFR and 3x 500 ul with fresh binding BFR (20 mM Hepes, 100 mM KCl, 0,05%, 1 mM DTT, complete protease inhibitors). For pull down experiments 50% bead slurry in binding BFR was prepared. GUVs tubulation studies PreScission protease was applied to beads and purified recombinant protein was eluted as described in manufacturers protocol (Ge Healthcare).

### GST Pull-Down

Expression of GST-tagged PSTPIP1, PSTPIP1 F-BAR domain only, PSTPIP1 SH3 domain only and PSTPIP2 in *E*. *coli*, was induced with 0.1 mM isopropyl-D-thiogalactoside (IPTG) for 20 h at 20°C. Protein was pulled down from bacterial lysate by glutathione-sepharose beads. Briefly: osteoclast lysates were incubated with glutathione-sepharose beads for 1 h at 4°C before they were added to the GST-tagged protein variants on glutathione-sepharose beads for 1 hr incubation at 4°C. Precipitates were washed 4 times with lysis buffer; proteins were eluted, and resolved by SDS-PAGE.

### Mass Spectrometry and Data Analysis

Coomassie stained protein bands were excised from the gel, cut into 1mm-cubes and washed twice with ultra-pure water to remove SDS. The gel pieces were then washed twice with 50% (v/v) acetonitrile (ACN) in 25mM ammonium bicarbonate (ABC) for 5min and shrunk by dehydration in ACN. The ACN was removed and the gel pieces were re- hydrated in 50mM ABC. After 5min the same volume of ACN was added for 5min and finally removed completely. The gel pieces were shrunk again in ACN for 5min, ACN was removed and gel pieces were dried in a vacuum centrifuge. Disulfide bonds were reduced by incubation with 10mM DTT in 100 mM ABC for 45min at 56°C. Alkylation was performed by replacing the DTT solution with 55mM iodoacetamide in 100 mM ABC. After 20min at 25°C in the dark, the gel pieces were washed with twice 50% (v/v) ACN in 25 mM ABC, shrunk by dehydration in ACN, and dried in a vacuum centrifuge. The gel pieces were incubated with 100 ng trypsin (sequencing grade, Promega) at 37°C overnight in 20 μl of 25mM ABC. To extract the peptides, 20 μl of 0.5% (v/v) trifluoroacetic acid (TFA) in ACN were added, the samples were sonicated and vortexed for 5min each. The supernatant was transferred into new tubes and the gel pieces were washed, sonicated and vortexed again with 20 μl ACN. The supernatants were combined and dried in a vacuum centrifuge. For mass spectrometric analysis of the peptide mixture, samples were re-dissolved in 5 μl 0.1% (v/v) TFA in water, referred as analyte solution.

Peptides were separated on an UltiMate3000 nanoHPLC system (Dionex, Amsterdam, The Netherlands) equipped with a PepMap C18 nano trap column (3mm, 100Å, 2cm x 75mm i.d.) and a PepMap C18 analytical column (3mm, 100Å, 15cm x 75mm i.d.) directly coupled to the nanoelectrospray source (Proxeon, Odense, Denmark) of a LTQ Orbitrap XL mass spectrometer (Thermo Fisher Scientific, Bremen, Germany). Peptides were eluted with an 80 min linear gradient of 5-45% acetonitrile in 0.1% formic acid at 200 nL/min. Mass spectra were acquired in a data-dependent mode with one MS survey scan (resolution of 60,000) in the Orbitrap and MS/MS scans of the eight most intense precursor ions in the LTQ. Data analysis was done using MaxQuant version 1.2.2.5 (Cox 2008). Peak lists were searched against a database containing 16,339 entries from the UniProt-KB/Swiss-Prot mouse database (release 2011_02) and 255 frequently observed contaminants as well as reversed sequences of all entries and the following search criteria: (i) enzyme specificity, trypsin; (ii) mass accuracy, 6 ppm and 0.5 Da for precursor ion and fragment ion mass tolerance, respectively; (iii) fixed and variable modifications, cysteine carbamidomethylation and methionine oxidation as well as modified arginine and lysine (for SILAC experiments), respectively; (iv) maximum of two missed cleavage sites. In SILAC experiments cells were cultured in DMEM lacking arginine (PAN Biotech) supplemented with 10% (vol/vol) dialyzed FCS. Arg-6 and Arg-0 SILAC media were prepared by adding l-arginine-U-^13^C_6_ (Cambridge Isotope Laboratories) or the corresponding nonlabeled amino acid, respectively, and cells were cultured for 5 days in SILAC media. Peptide identifications were accepted based on their posterior error probability until less than 1% reverse hits were retained while protein false discovery rates were < 1%. Proteins were considered if at least two peptides were identified. SILAC-based protein quantification was performed by MaxQuant based on the median SILAC ratios of at least two peptides per sample. Results were only included if the experiment-to-experiment variation of protein ratios were <30% in two independent experiments with SILAC label swapping.

### GUVs Tubulation Assays

Giant unilamellar vesicles (GUVs) were generated by electro-swelling as described earlier (Anitei et al., 2011). Briefly: 6 μl of temperated lipid mixture (1 mg/ml of 5% PIP 4,5, 5%PI, 10% PS, 10% PE, 35% Cholesterol, 10% SM, 25% PC) was pipeted as small drops onto conductive surface of electrode chamber. Assembled chamber heated for 50°C filled with sucrose at osmolarity 294 mOsm/Kg were subjected to current at frequency of 10 GHz for 1.5 hr and then decreased to 26 Hz for 2 hrs.

Microscopy chambers Lab-TEK II ((Nunc, Langenselbold, Germany) were blocked 2 hrs with 2% BSA. Washed 3x with 200 μl of BFR (BFR 10 mM Hepes, 150 mM NaCl, 295 mOsm/Kg). To each chamber 20 μl of BFR was added. 0.5 μl of GUVs were transferred by use of cut pipet tip into isoosmolar BFR and let settle for 5 min at RT. Proteins were added to chambers with GUVs to final concentration 1,5 μM. Osmolarity was increase by addition of 10 ul of 750 mOsm/Kg sucrose to obtain final 400 mOsm/Kg. GUVs were than incubated for 15 min at RT and subjected to inverted LSM 510 META confocal microscope equipped with a 40x, 1.2 numerical aperture water-immersion objective.

### Resorption Assays

For resorbing assay, osteoclasts were RNAi depleted for PSTPIP1, PSTPIP2, PSTPIP1/2, PTPN6, PTPN12, PTPN22, SHIP1, SHIP2, SHIP1/2, and Src and plated on BioCoat osteological^™^ discs. After 48hrs of RNAi, Osteoclasts were detached from osteological^™^ discs by adding 1mL of detaching solution (of ~6% NaOCl and ~5,2% NaCl). Cells were shacked on a shaker after 5min and washed 2x with H2O. Osteological^™^ discs were rinsed 2x with H2O and dried. Osteological^™^ discs without osteoclasts were imaged with Zeiss SteREO Discovery.V20 and the resorbed area determined with FIJI software.

### Generation of Mouse B6.129-Pstpip1tm1Spg/J +/+ Ctsk-CreE2+/- Strain

Two male mice of the strain B6.129-Pstpip1tm1Spg/J were purchased from Jackson Laboratory. Mice were housed and rederived at the MPI-CBG, Dresden, Germany. In short, males were mated with superovulated C57BL/6J females in quarantine. Pre-implantation stage embryos were collected, washed and transferred into specific pathogen free (SPF) pseudo-pregnant C57BL/6J females, housed at the pathogen free area. Resultant B6.129-Pstpip1tm1Spg/J +/- F1 mice were then mated with their B6.129-Pstpip1tm1Spg/J +/- F1 siblings4 to obtain B6.129-Pstpip1tm1Spg/J +/+ mice. These mice were mated with CtsK-CreE2 mice to create B6.129-Pstpip1tm1Spg/J +/+ Ctsk-CreE2+/- mice and were then used for experiments. All animal studies were performed in strict accordance with German Animal Welfare legislation. All protocols were approved by the Institutional Animal Welfare Officer (Tierschutzbeauftragter), and necessary licenses were obtained from the regional license granting body (Landesdirektion Dresden, Germany; permit numbers: 24–9168.24-9/2009-1 and 24–9168.11-9/2010-3).

### Cre Recombination of PSTPIP1 in Osteoclasts

Bone marrow cells of 8 to 10 weeks old B6.129-Pstpip1tm1Spg/J +/+ Ctsk-CreE2+/- were sacrificed by CO_2_ asphyxiation to isolate long bones (femur and tibia). Femurs and tibias were cleaned of the surrounding soft tissue. Following excision of the ends of the long bones, bone marrow was removed by flushing with aMEM medium. Cells were then differentiated into osteoclasts or macrophages by respective addition of 20 ng/ml Macrophages Colony Stimulating Factor (MCSF, PeproTech, germany) and 50 ng/ml recombinant soluble Receptor activator of nuclear factor kappa-B ligand (RANKL, PeproTech, germany). Cells were grown in aMEM medium supplemented with 10% FCS, 2 mM HEPES, 1% penicillin/streptomycin and 1% L- glutamine in 10% CO2, 95% humidity at 37°C. After differentiation, 4-OHT (10^-3^ M) diluted in ethanol was added to the cultured cells.

## Results

### PSTPIP1 and PSTPIP2 Regulate Podosome and Sealing Zone Dynamics

PSTPIP1/2 amino acid sequences predict that they contain putative F-BAR domains. Such domains have the property to promote membrane tubulation both *in vitro* and *in vivo* [10, 11]. In agreement with others [9], the incubation of recombinant PSTPIP1/2 with giant unilamellar vesicles led to the formation of membrane tubules (S1A Fig) and the over-expression of GFP-tagged PSTPIP1 or PSTPIP2 in HEK cells also led to membrane tubule formation (S1B Fig). Thus, although their crystal structure was not yet established, PSTPIP1/2 exhibited the typical properties of *bona fide* BAR-domain containing proteins.

**Fig 1:**
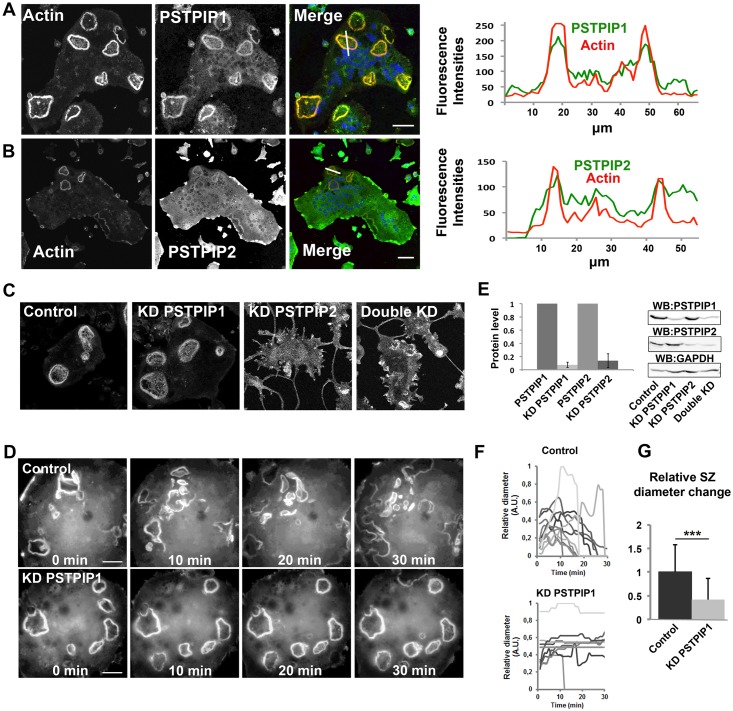
PSTPIP1/2 localization and role in sealing zone dynamics. **A, B** Osteoclasts grown on osteological discs were fixed and stained with antibody against PSTPIP1 (green) (**A**) or PSTPIP2 (green) (**B**) and phalloidin (red). (scale bars: 20μm) and images were analyzed using the Fiji software. Fluorescence intensities across the indicated white lanes are indicated and Pearson’s coefficients were calculated (0.65 for PSTPIP1 and 0.25 for PSTPIP2 from datasets of three different experiments N = 3, and n = 68 measurements). **C** Effect of siRNA-mediated depletion of PSTPIP1 or PSTPIP2 or both PSTPIP1 and PSTPIP2 on sealing zone assembly. Osteoclasts were treated with siRNA targeting the indicated genes and then grown for 48 hours on osteological discs as indicated in Materials and Methods. Cells were then fixed and stained with phalloidin. Scale bars 20 μm. **D** Sealing zone dynamics in PSTPIP1-depleted osteoclasts. Osteoclasts were treated with siRNAs targeting PSTPIP1 and then plated on osteological discs. After 24 hours, they were infected with a recombinant adenovirus encoding the mRFP-Ezrin actin-binding domain. After 32 hours, osteoclasts were observed by time-lapse videomicroscopy (300 msec. per frame, 1 frame per 1 min., see S1 and S2 Movies 1, 2). **E** The knockdown efficiencies were determined by western blotting and quantified. The figures presented are representative of at least 3 independent experiments (mean ± SD). **F** Sealing zone diameter was measured using the Fiji software. The relative sealing zone diameter (biggest sealing zone as reference) was plotted for each sealing zone assessed (n = 3 independent experiments). **G** The change of relative sealing zone diameter per minute was plotted and tested using students t-test. (mean ± SD * represents p<0.05 and ** p<0.01, *** p< 0.001).

To investigate PSTPIP1/2 functions, we first localized them in Raw.267-derived osteoclasts grown on osteological discs (hydroxyapatite-coated surfaces). Both endogenous PSTPIP1 and PSTPIP2 localized to actin-rich sealing zones (Fig 1A and 1B). A mRFP-tagged PSTPIP2 expressed in Raw.267-derived osteoclasts also localized to actin rich sealing zones (S2 Fig). Whereas the siRNA-mediated PSTPIP2 depletion (90% efficiency without affecting PSTPIP1 expression, Fig 1E) led to a complete disappearance of podosomes and sealing zones (Fig 1C), the siRNA-mediated PSTPIP1 depletion (>90% efficiency without affecting PSTPIP2 expression, Fig 1E) did not produce any apparent effects on sealing zones observed in fixed osteoclasts (Fig 1C). However, the examination of PSTPIP1-depleted osteoclasts using time-lapse video microscopy revealed that their sealing zones, labeled by the mRFP-tagged actin-binding domain of Ezrin (mRFP-ABDE), had significantly lost their dynamic state when compared to control osteoclasts (Fig 1D, 1F and 1G, S1 and S2 Movies). These results are in agreement with the observation that PSTPIP1 depletion in macrophages stabilizes podosomes [15]. Similar to PSTPIP2 depletion, the depletion of both PSTPIP1 and PSTPIP2 abolished podosome and sealing zone formation (Fig 1C). These results suggest that PSTPIP2 regulates podosome and sealing zone assembly, whereas PSTPIP1 regulates their disassembly.

**Fig 2:**
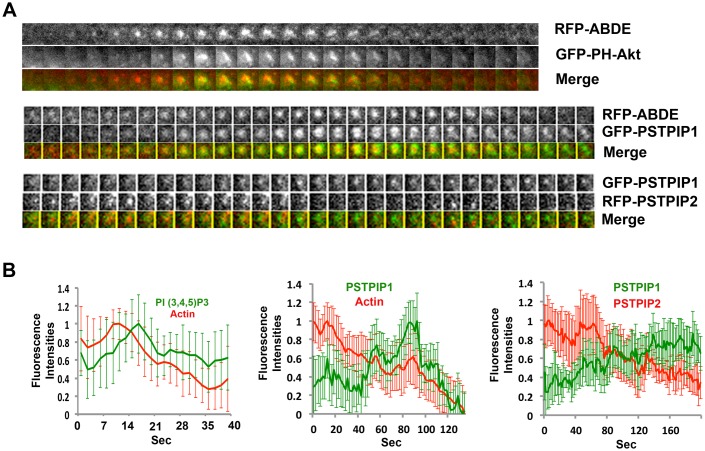
PSTPIP1/2 dynamics at podosomes. Osteoclasts were grown on glass coverslips and transfected with constructs to express mRFP-tagged actin-binding domain of Ezrin (mRFP-ABDE) together with the GFP-PI3,4,5 P3 binding domain of Akt kinase or mRFP-Ezrin actin-binding domain and GFP-PSTPIP1 or GFP-PSTPIP1 and mRFP-PSTPIP2. **A** Individual podosomes were then observed by time-lapse videomicroscopy 48 hours after transfection (100 msec. per frame, 1 frame per 2 sec., S3, S4 and S5 Movies). **B** The fluorescence intensities represent the variations of mean fluorescence intensity associated to 35 podosomes in three independent experiments.

To test this hypothesis, we used time-lapse videomicroscopy to analyze podosome dynamics. Osteoclasts were grown on glass surfaces that allow podosome assembly, but prevent their packing into sealing zones. Podosomes labeled with mRFP-ABDE had a half-life of 2–4 minutes, as previously described [3], and their assembly/disassembly correlated with the synthesis and turnover of PI(3,4,5)P3, detected with the GFP-PH domain of Akt (Fig 2A and 2B, S3 Movie). PSTPIP1 and PSTPIP2 exhibited different dynamics. PSTPIP2 was always detected on already formed F-actin-rich podosomes, which disassembled while PSTPIP1 was recruited. (Fig 2A and 2B, S4 and S5 Movies). Altogether, these results indicate that PSTPIP1 and PSTPIP2 have opposite roles in podosome dynamics. PSTPIP2 controls podosome assembly whereas PSTPIP1 substituting PSTPIP2 at podosomes controls podosome disassembly.

### PSTPIP1/2 Interactomes

To better understand PSTPIP1/2 function, we then identified their interactors. For this, we first performed pull-down experiments using recombinant PSTPIP1, its F-BAR, or SH3 domains fused to GST or recombinant GST-PSTPIP2, as baits, and osteoclast lysates as a source of proteins. Pulled-down proteins were identified using semi-quantitative mass spectrometry based on MS2 spectrum counting. We identified ≈150 putative PSTPIP1 interactors binding either to its F-BAR or SH3 domain, including several known PSTPIP1 interactors and podosomal components (Table 1). These were classified into several functional groups. The first group comprised phospho-tyrosine protein phosphatases of the PEST family such as PTPN12, PTPN18, PTPN22 and PTPN6 that bound to the F-BAR domain. The second group comprised tyrosine protein kinases such as Syk and BTK binding to the F-BAR domain, and Abl1/2 binding to the SH3 domain. A third group, mostly interacting with the PSTPIP1 SH3 domain, comprised the GTPase dynamin (dynamin2), several ARF or Rho GTPase activating proteins (GAPs) including GIT1, ARAP1 and ASAP1, Rho-GAPs (RHG17), and the Rho Guanine exchange factors DOCK 5 and 8. A fourth group comprised of several actin-nucleation promoting factors such as WASP, VASP, WASL and IQGAP1, and the actin motors Myosin-9 and Myosin-II. Finally, the phosphoinositol phosphate 5-phosphatases SHIP1/2 and synaptojanin bound to the PSTPIP1 SH3 domain.

**Table 1:** PSTPIP1 interactors.

Protein name	Gene name	Acc. number	Domain	Counts
*Phosphatases*				
Tyrosine-protein phosphatase non-receptor type 12	PTN12	P35831	BAR	624
Tyrosine-protein phosphatase non-receptor type 18	PTN18	Q61152	BAR	12
Tyrosine-protein phosphatase non-receptor type 22	PTN22	P29352	BAR	49
Tyrosine-protein phosphatase non-receptor type 6	PTN6	P29351	BAR	54
*Kinases and interactors*				
Tyrosine-protein kinase ABL1	ABL1	P00520	SH3	16
Tyrosine-protein kinase ABL2	ABL2	Q4JIM5	SH3	499
Tyrosine-protein kinase BTK	BTK	P35991	BAR	46
Tyrosine-protein kinase SYK	KSYK	P48025	BAR	12
KH domain-containing protein 1	KHDR1	Q60749	SH3	459
SH3 domain-binding protein 1	3BP1	P55194	SH3	1383
SH3 domain-containing kinase-binding protein 1	SH3K1	Q8R550	SH3	2005
*GTPases*, *GEFs*, *GAPs*				
ARF GTPase-activating protein GIT1	GIT1	Q68FF6	BAR	38
Arf-GAP, ANK and PH domain-containing protein 1	ARAP1	Q4LDD4	SH3	1642
Arf-GAP, SH3, ANK, PH domain-containing protein 1	ASAP1	Q9QWY8	SH3	161
Dedicator of cytokinesis protein 5	DOCK5	B2RY04	SH3	2968
Dedicator of cytokinesis protein 8	DOCK8	Q8C147	SH3	889
Dynamin-1	DYN1	P39053	SH3	66
Dynamin-2	DYN2	P39054	SH3	5393
Dynamin-3	DYN3	Q8BZ98	SH3	169
Ras and Rab interactor 3	RIN3	P59729	SH3	1548
Ras GTPase-activating protein-binding protein 1	G3BP1	P97855	BAR	246
Ras GTPase-activating-like protein IQGAP1	IQGA1	Q9JKF1	BAR	37
Rho GTPase-activating protein 17	RHG17	Q3UIA2	SH3	1241
*PIPs phosphatases*				
Pl(3,4,5)P3 5-phosphatase 1	SHIP1	Q9ES52	SH3	1362
Pl(3,4,5)P3 5-phosphatase 2	SHIP2	Q6P549	SH3	61
Synaptojanin-1	SYNJ1	Q8CHC4	SH3	1967
*Cytoskeleton*				
CD2-associated protein	CD2AP	Q9JLQ0	SH3	544
Engulfment and cell motility protein 1	ELMO1	Q8BPU7	SH3	1425
Microtubule-actin cross-linking factor 1	MACF1	Q9QXZ0	BAR	17
Myosin-9	MYH9	Q8VDD5	BAR	454
Myosin-If	MYO1F	P70248	BAR	79
Neural Wiskott-Aldrich syndrome protein	WASL	Q91YD9	SH3	152
Plectin	PLEC	Q9QXS1	BAR	704
Vasodilator-stimulated protein	VASP	P70460	SH3	408
WAS/WASL-interacting protein 1	WIPF1	Q8K1I7	SH3	3661
Wiskott-Aldrich syndrome protein	WASP	P70315	SH3	1831
*Ubiquitination*				
E3 ubiquitin-protein ligase CBL	CBL	P22682	SH3	241
E3 ubiquitin-protein ligase CBL-B	CBLB	Q3TTA7	SH3	576

Recombinant GST-PSTPIP1, GST-SH3, and GST-F-BAR were incubated with osteoclast lysates. Bound proteins were isolated on Glutathione beads, resolved by SDS-PAGE and identified by semi-quantitative mass spectrometry analysis based on MS2 spectral counting. Protein names, gene names, and accession numbers are indicated. Proteins interacting with PSTPIP1 domains are indicated by BAR or SH3. MS2 spectral counts are represented in the last column.

We also established the PSTPIP2 interactome (S1 Table). Compared to PSTPIP1, fewer PSTPIP2 interactors were identified, as expected from the fact that PSTPIP2 lacks a SH3 domain. Interestingly, several proteins (PTPN12, PTPN22 and IQGAP1) interacted with both PSTPIP1 and PSTPIP2. Other proteins exclusively bound the F-BAR domain of either PSTPIP1 (e.g. PTPN6) or PSTPIP2 (e.g. Talin-1). The same interactors were found by label-free quantitative proteomics comparing GST-tagged PSTPIP1 and GST-tagged PSTPIP2 interactomes (data not shown). We confirmed several of these in vitro interactions by immunoprecipitating endogenous PSTPIP1/2 from osteoclast lysates with specific antibodies, followed by western blotting using specific antibodies against selected interactors (e.g. PTPN6 and SHIP1/2) (S3 Fig). In particular, we confirmed that PSTPIP1, PTPN6 and SHIP1/2 formed a complex in osteoclasts.

### PTPN Phosphatases and Podosome/Sealing Zone Dynamics

**Fig 3:**
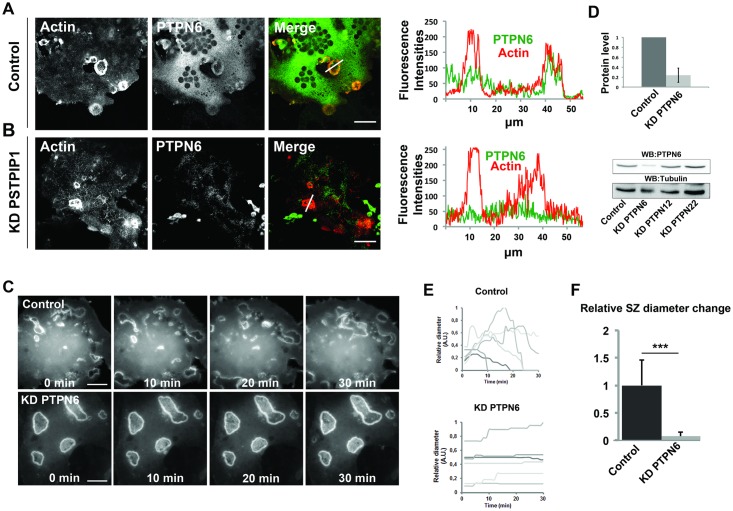
PTPN6 localization and effect of its depletion on sealing zone dynamics. **A** Osteoclasts grown on osteological discs were fixed and stained with PSTPIP1 antibodies (green) and phalloidin (red). (scale bars: 20μm). **B** PSTPIP1-depleted osteoclasts were treated similarly (scale bars: 20μm) Images were analyzed using the Fiji software. Fluorescence intensities across the indicated white lanes are indicated and Pearson’s coefficients were calculated (0.35 for PTPN6 from datasets of three different experiments N = 3, and n = 37 measurements). **C** Sealing zone dynamics in PTPN6-depleted osteoclasts. Osteoclasts were treated with siRNAs targeting PTPN6 and then plated on osteological discs. After 24 hours, they were infected with a recombinant adenovirus encoding the mRFP-Ezrin actin-binding domain. After 32 hours, osteoclasts were observed by time-lapse videomicroscopy (100 msec. per frame, 1 frame per 1 min., see S6 and S7 Movies). **D** The knockdown efficiencies were determined by western blotting and quantified. The figures presented are representative of at least 3 independent experiments. Scale bars 20 μm (mean ± SD). **E** Sealing zone diameter was measured using the Fiji software. The relative sealing zone diameter (biggest sealing zone as reference) was plotted for each sealing zone assessed. **F** The change of relative sealing zone diameter per minute was plotted and tested using students t-test. (mean ± SD * represents p<0.05 and ** p<0.01, *** p< 0.001).

To investigate the biological significance of this PSTPIP1, PTPN6 and SHIP1/2 complex, we first examined PTPN6 localization and function. PTPN6 localized to sealing zones (Fig 3A). Its localization was lost upon PSTPIP1 depletion (Fig 3B), as expected for a specific PSTPIP1 interactor. To establish its functional importance, we examined by time-lapse video microscopy the dynamics of mRFP-ABDE-positive sealing zones in PTPN6-depleted osteoclasts (90% knockdown efficiency without affecting PTPN12 or PTPN22 expression, Fig 3D). Fig 3C (S6 and S7 Movies) shows that PTPN6-depleted osteoclasts grown on osteological discs significantly lost sealing zone dynamics (Fig 3E and 3F), thus fully recapitulating PSTPIP1 depletion.

We also examined the localization and function of PTPN12 and PTPN22. Although they were detected at sealing zones, their localization was not affected by PSTPIP1 depletion, as expected from proteins also interacting with PSTPIP2 (S4A Fig). We therefore determined how PTPN12- and PTPN22-depletion could affect sealing zone dynamics using time-lapse video microscopy in osteoclasts expressing the mRFP-tagged actin-binding domain of Ezrin. Their depletion (95–90% efficiency, S4C Fig) gave rise to phenotypes resembling PSTPIP2 depletion. Only rare and small, quickly collapsing, sealing zones could be observed in PTPN12- or PTPN22-depleted osteoclasts (S4D and S4E Fig, S8–S10 Movies). This indicated that the main function of these PSTPIP2 interactors was to regulate podosome and sealing zone assembly, as did PSTPIP2.

### PIP3, 5 Phosphatases SHIP1/2 and Podosome/Sealing Zone Dynamics

**Fig 4:**
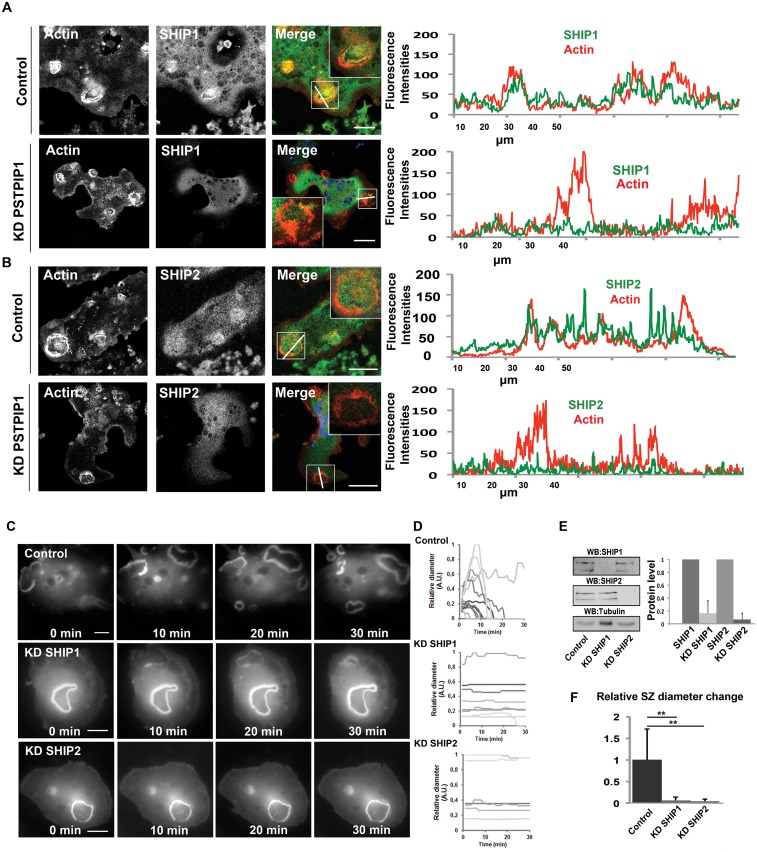
SHIP1/2 localization and effect of their depletion on sealing zone dynamics. **A, B** Non treated osteoclasts or osteoclasts treated with siRNAs targeting PSTPIP1 were grown on osteological discs, then fixed and stained with anti SHIP1 **(A)** or SHIP2 antibodies (**B)** (green) and phalloidin (red) (scale bars: 20 μm) Images were analyzed using the Fiji software. Fluorescence intensities across the indicated white lanes are indicated, magnifications of encircled regions were inserted and Pearson’s coefficients were calculated (0.49 for SHIP1, 0.24 for SHIP2 from datasets of three different experiments N = 3, and n = 103 for SHIP1, and n = 130 for SHIP2 measurements). **C** Sealing zone dynamics in SHIP1/2 depleted osteoclasts. Osteoclasts were treated with siRNAs targeting SHIP1 or SHIP2 and then plated on osteological discs. After 24 hours, they were infected with a recombinant adenovirus encoding the mRFP-Ezrin actin-binding domain. After 32 hours, osteoclasts were observed by time-lapse videomicroscopy (100 msec. per frame, 1 frame 1 min., see S11–S13 Movies). **D** Sealing zone diameter was measured using the Fiji software. The relative sealing zone diameter (biggest sealing zone as reference) was plotted for each sealing zone assessed. **E** The knockdown efficiencies were determined and quantified by western blotting. The figures presented are representative of at least 3 independent experiments (mean ± SD). Scale bars 20 μm. **F** The change of relative sealing zone diameter per minute was plotted and tested using students t-test. (mean ± SD * represents p<0.05 and ** p<0.01, *** p< 0.001)

We then reasoned that the protein-tyrosine phosphatase PTPN6 bound to the F-BAR domain of PSTPIP1 could regulate the phosphorylation state and the activity of proteins bound to the SH3 domain of PSTPIP1. To identify PTPN6 substrates, we performed SILAC (Stable Isotope Labeling with Amino acid in cell Culture) experiments to evaluate the phosphotyrosine-containing proteomes of osteoclasts treated with phenylarsine oxide (PAO), a membrane permeable PTPN inhibitor [16]. Lysates of osteoclasts treated or not with this inhibitor were mixed (equal volumes) and immunoprecipitated with anti-phosphotyrosine antibodies. The immununoprecipitates were then analyzed by quantitative mass spectrometry. Among the ≈350 proteins identified, ≈150 proteins exhibited changes in their tyrosine phosphorylation state (Table 2). Among these we found the PSTPIP1 SH3 domain interactors SHIP1, ABL1, GIT1, VASP, as well as other proteins critical for podosome/sealing zone assembly, such as the protein-tyrosine kinase Pyk2, the focal adhesion kinase FAK1 and paxillin. We confirmed that PTPN6 or PSTPIP1 knockdown results in a higher phosphorylation state of tyrosine residues in SHIP1/2 (S5 Fig). In osteoclasts grown on osteological discs, the PIP3, 5 phosphatases SHIP1 and SHIP2 were detected at sealing zones and, to a small extent, at the ruffled border (Fig 4A). Their localization at sealing zones was reduced upon PSTPIP1 knockdown (Fig 4A). We then examined, using time-lapse videomicroscopy, the phenotype of osteoclasts expressing the GFP-tagged ezrin actin-binding domain and depleted of either SHIP1 or SHIP2. Fig 4C, 4D and 4F (S11–S13 Movies) shows that the siRNA-mediated depletion of SHIP1 or SHIP2 led to the formation of sealing zones with significantly reduced dynamic states, thus phenocopying PSTPIP1 or PTPN6 depletion. This illustrates the complementary functional importance of SHIP1/2 and suggests that SHIP1/2-dependent, PIP(3,4,5)P3 turnover at podosomes and sealing zones is regulated by PTPN6 bound to the PSTPIP1-BAR domain.

**Table 2:** Effect of PAO on tyrosine phosphorylation.

Protein name	Gene	Acc Nb.	SILAC ratio
*PIPs phosphatases*			
Pl(3,4,5)P3 5-phosphatase 1	SHIP1	Q9ES52	0.1
*Cytoskeleton*			
PSTPIP1	PSTPIP1	P97814	0.54
Paxillin	PAXI	Q8VI36	0.06
Tensin-3	TENS3	Q5SSZ5	0.08
ARF GTPase-activating protein GIT1	GIT1	Q68FF6	0.22
Wiskott-Aldrich syndrome protein WAVE-2	WASF2	Q8BH43	0.17
Alpha-actinin-4	ACTN4	P57780	0.49
Focal adhesion kinase 1	FAK1	P34152	0.39
Protein-tyrosine kinase Pyk2	FAK2	Q9QVP9	0.06
Myosin-Id	MYO1D	Q5SYD0	0.13
Myosin-Ig	MYO1G	Q5SUA5	0.49
Myosin-14	MYH14	Q6URW6	0.06
Filamin-A	FLNA	Q8BTM8	0.17
Filamin-B	FLNB	Q80X90	0.47
Filamin-C	FLNC	Q8VHX6	0.15
*Proton pumps*			
V-type proton ATPase subunit A	VATA	P50516	0.53
V-type proton ATPase subunit B	VATB2	P62814	0.52
V-type proton ATPase subunit C 1	VATC1	Q9Z1G3	0.37
V-type proton ATPase subunit D	VATD	P57746	0.55
V-type proton ATPase subunit E 1	VATE1	P50518	0.47
V-type proton ATPase subunit H	VATH	Q8BVE3	0.51
*Others*			
Abl interactor 1	ABI1	Q8CBW3	0.36
Calreticulin	CALR	P14211	0.49
Src kinase-associated phosphoprotein 2	SKAP2	Q3UND0	0.49

Raw264.7 cells were grown with heavy and light amino acids as indicated in materials and methods. They were then differentiated into osteoclasts with RANKL. Osteoclasts were then incubated or not with 1 μM PAO during 45 min. Control and PAO treated osteoclasts were lysed, the corresponding lysate were mixed in a 1:1 ratio, the phosphotyrosine containing proteins were immunoprecipitated with anti phosphotyrosine antibodies and resolved by SDS PAGE. Protein identification and quantitative changes (SILAC ratio) are described in materials and methods. Protein names, gene names, accession numbers, and SILAC ratios are indicated. A 0.85 ratio reflects an unchanged phosphorylation state.

### Podosome/Sealing Zone Dynamics and Bone Degradation

**Fig 5:**
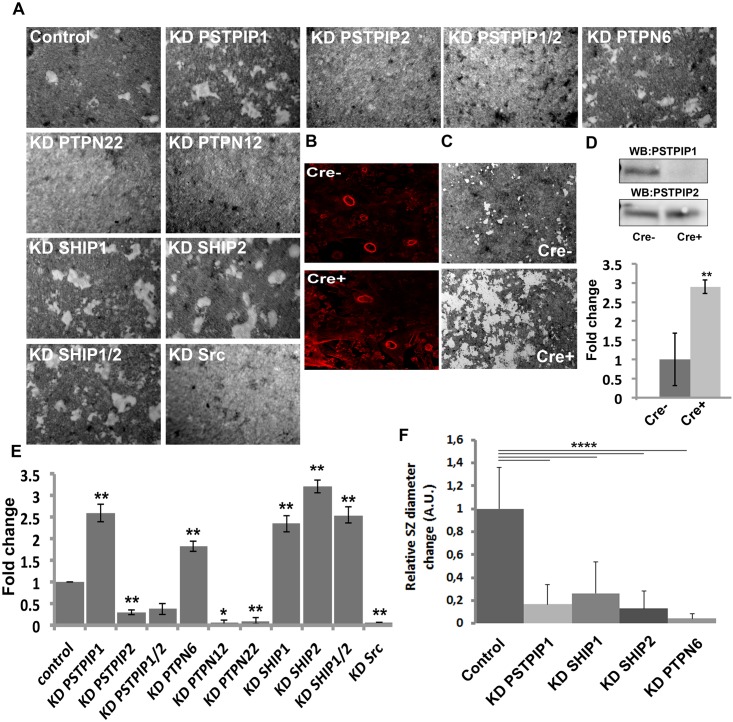
Sealing zone dynamics and osteoclast activity in digestion. **A** Osteoclasts were treated with siRNAs targeting the indicated genes and then plated onto osteological discs. After 48 hours, the digested areas of osteological discs (seen in white whereas the surface appears in grey) were visualized by microscopy. **B** Bone marrow osteoclast precursors isolated from long bones of Cre-ERT2+/+,PSTPIP1+/+ and Cre-ERT2-/-,PSTPIP1+/+ mice were treated with M-CSF and RANKL and the resulting osteoclasts were further treated with tamoxifen as indicated in Materials and Methods. Sealing zones of osteoclasts plated on osteological discs were stained with phalloidin and observed by confocal microscopy. **C** PSTPIP1 and PSTPIP2 expression was determined by western blotting. **D** The activity of these osteoclasts in resorption was determined as indicated above and as described in Materials and Methods. Scale bars 50 μm. The figures presented are representative of at least 3 independent experiments. **E** Quantification of resorption pit assays were perfomed as indicated in materials and methods. Quantifications of areas from 3 different experiments are plotted in chart (mean ± SD * represents p<0.05 and ** p<0.01, significance was calculated using *t*-test). Knockdown efficiencies (>90%) determined by western blotting were as presented in previous figures. **F** The change in relative sealing zone diameter per minute was plotted for each condition; controls of individual experiments were taken together. Statistical significance was tested using students t-test for each KD. (Mean ± SD, **** p< 0.0001).

In osteoclasts, the inability to assemble podosomes and sealing zones impairs bone degradation. However, it is unknown how changes in podosome and sealing zone dynamics affect osteoclast activity in bone degradation. To address this question, we examined the capability of osteoclasts depleted of PSTPIP1, PTPN6 or SHIP1/2, which reduced sealing zone dynamics (see Fig 5F), to digest osteological discs mimicking bone surfaces. Fig 5A shows that osteoclasts depleted of any of these components exhibited a 2–3 fold higher capacity of digesting the surface of osteological discs. In contrast, osteoclasts depleted of PSTPIP2, PTPN12, PTPN22 or Src, that could not assemble podosomes and sealing zones were also unable to digest such surfaces. We also evaluated the ability of mouse primary PSTPIP1-/- osteoclasts to digest osteological discs. For this, mice carrying a floxed PSTPIP1 gene were crossed with mice expressing Cre-ERT2 under the control of the Cathepsin K promoter that allows performing tamoxifen-induced conditional knockouts in osteoclasts [17]. Cre-ERT2-positive and negative mice with a floxed PSTPIP1 gene were generated, and primary osteoclasts were obtained after the differentiation of their bone marrow precursors with M-CSF and RANKL. The treatment of these primary osteoclasts with tamoxifen resulted in an efficient knockout of PSTPIP1, without affecting PSTPIP2 expression (Fig 5C). Tamoxifen treatment of Cre-ERT2+/+, PSTPIP1+/+ and Cre-ERT2-/-, PSTPIP1+/+ osteoclasts did not affect their ability to form sealing zones (Fig 5B). However, it increased (≈3 fold) the capacity of Cre-ERT2+/+, PSTPIP1+/+ osteoclasts to digest the surface of osteological discs (Fig 5D). Altogether, these results indicate that the dynamic instability of podosomes and sealing zones controlled by PSTPIP1/PTPN6/SHIP1/2 complex is key in modulating the activity of osteoclasts in bone digestion.

## Discussion

**Fig 6:**
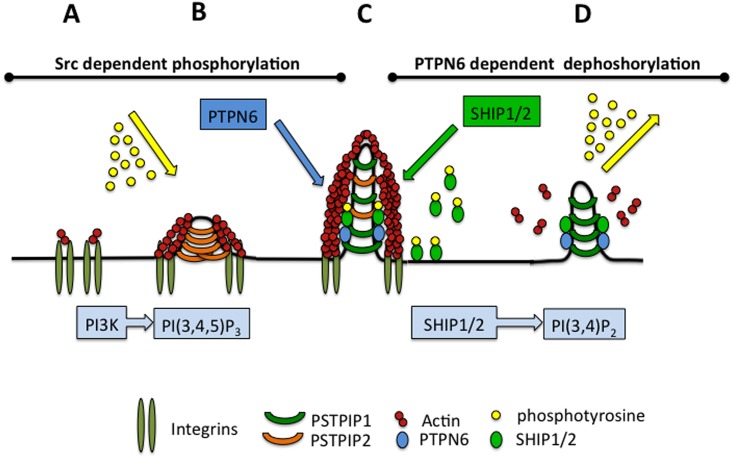
Proposed function of PSTPIP1/2 and interactors in podosome and sealing zone dynamics. **A** Podosome assembly is initiated by integrin engagement triggering PI(3,4,5)P3 synthesis and Src-dependent phosphorylation of podosomal components. **B** PSTPIP2, acting as a membrane scaffolding protein, initiates membrane tubulation. PSTPIP2 recruit podosomal components such as Talin, which connects integrins with actin cytoskeleton. This step also involves PTPN12 and PTPN22. **C** PSTPIP1, also sensing membrane curvature, substitutes PSTPIP2 by a yet unknown mechanism. PSTPIP1 recruits interactors, in particular PTPN6 and SHIP1/2. **D** PTPN6 can dephosphorylate several podosomal components and regulate their activity, in particular SHIP1/2 to turnover PI(3,4,5)P3 into PI(3,4)P2. PI(3,4,5)P3 turnover combined with dephorylation of Src substrates would destabilize the overall podosome architecture. Podosome and sealing dynamics would therefore rely on mechanisms coordinating PI(3,4,5)P3 synthesis/turnover, membrane scaffolding properties of the F-BAR PSTPIP1/2 and the Src- and PTPN6-dependent phosphorylation state of podosomal components.

Our study illustrates the essential function of PSTPIP1 and PSTPIP2 in podosome and sealing zone dynamics in osteoclasts. Remarkably, these F-BAR-domain proteins exhibit opposite activities. PSTPIP2, acting as a membrane scaffold, is essential for podosome and sealing zone assembly. PSTPIP1, substitutes PSTPIP2 on mature podosomes, and regulates podosome and sealing zone disassembly. PSTPIP1 recruits through its F-BAR domain the protein tyrosine phosphatase PTPN6 that can dephosphorylate and regulate the activity of podosome components bound to its SH3 domain, as illustrated for the PI(3,4,5)P3 5-phosphatases SHIP1/2. These results provide a mechanism by which the PSTPIP1/PTPN6/SHIP1/2 complex regulates the dynamic instability of podosomes assembled upon Src-dependent phosphorylation and PI(3,4,5)P3 signalling. In addition, it shows that this dynamic assembly/disassembly of podosomes and sealing zones is key for osteoclast activity in bone degradation (Fig 6).

PSTPIP1 and PSTPIP2 behave as typical F-BAR domain containing proteins sensing positive membrane curvature able to generate membrane tubules in vitro [10, 18, 19]. Previous studies have proposed that podosomes and invadopodia of cancer cells are protrusive structures of the plasma membrane [6, 20, 21]. Some others have proposed that podosomes of Rous sarcoma virus (RSV) transformed cells contain an invaginated tubular membrane in their core [22, 23] that contains actin surrounded by 250–500 nm rings containing two podosomal components, vinculin and talin [4]. Our data showing that PSTPIP2, a curvature-sensing protein, is essential for podosome assembly in osteoclasts would be consistent with a structural model in which podosomes contain an invaginated membrane tubule in their core. This structure would also explain the role that dynamin, a SRC-dependent GTPase that assembles on tubulated membranes during clathrin-mediated endocytosis, plays in podosome dynamics [24].

Our study illustrates the critical role of PSTPIP2 in podosome formation in mature Raw cell-derived osteoclasts since its knockdown prevents the formation of these structures. This finding would drastically differ from other studies showing that PSTPIP2 is a negative regulator of actin polymerization in undifferentiated Raw cells [25]. Whereas additional studies would be required to clarify this apparent discrepancy, it is possible that Src-dependent tyrosine phosphorylation of PSTPIP2 in mature osteoclasts modify the properties of this BAR-domain containing protein. Our study also shows that PSTPIP1 can substitute PSTPIP2 on mature podosomes, a phenomenon that has never been described for BAR-domain proteins. The mechanism underlying this switch remains unknown. Src-dependent phosphorylation may regulate this switch. Our study demonstrates that PSTPIP1 and PSTPIP2 play essential but opposite roles in podosome dynamics. Whereas PSTPIP2 regulates podosome and sealing zone assembly, PSTPIP1 regulates their disassembly. This dynamic instability of podosomes is important for sealing zone dynamics in osteoclasts. The arrangement of podosomes in higher ordered structures depends on the surface onto which cells adhere [3, 5, 6]. Podosomes assemble when osteoclasts are grown on glass, but condense into podosomal sealing zones when they are grown on bone [24] or on bone mimicking surfaces, such as osteological discs [3]. Sealing zone expansion and shrinking is allowed by a continuous assembly of podosomes at its outer rim, and a disassembly of podosomes in its inner rim [3]. Whereas this dynamic instability of podosomes and sealing zones regulate osteoclast activity in bone degradation, it may also play a role during other phases of bone digestion, i.e. the subsequent steps of cell adhesion and cell migration.

Besides acting as membrane scaffolds, PSTPIP1/2 are also used as docking platforms that recruit podosome components in osteoclasts. PSTPIP2 binds talin1, which connects αvβ3 integrins and F-actin, and is essential for bone degradation [26]. IQGAP1, an actin nucleation promoting factor essential for podosome assembly [27] and the Rac1 Guanine exchange factor DOCK5, which is essential for sealing zone assembly and bone degradation [28]. PSTPIP2 is also used as a docking platform by the tyrosine-protein phosphatases PTPN12 and PTPN22, which, together with PSTPIP2, contribute to stabilize podosomes and sealing zones in osteoclasts.

PSTPIP1 activity in podosome and sealing zone dissassembly is mediated by proteins interacting with its BAR or SH3 domain. Interestingly, PTPN6 specifically interacts with the PSTPIP1 BAR domain, although this exhibits a 60% homology with the BAR domain of PSTPIP2. In the future, a detailed analysis to map the PTPN6 binding sites on the PSTPIP1 BAR domain could help to better understand this interaction. Nevertheless, PTPN6 localization at sealing zones was strikingly dependent upon the presence of PSTPIP1. PTPN6 controls osteoclast resorption. In other cell types, such as macrophages [29] and neutrophils [30], PTPN6 regulates integrin-mediated adhesion. When PTPN6 function is impaired, these cells are hyper-adhesive and respond to integrin engagement more strongly than wild-type cells. PTPN6 contains two SH2 domains and a phosphatase domain. The N-terminal SH2 domain functions as an auto-inhibitory domain that blocks the catalytic domain in the ligand-free close conformation, in a phosphorylation-dependent manner [31]. In its open conformation, the PTPN6 phosphatase domain might regulate the phosphorylation state and the activity of proteins binding either to the SH2 domain of PTPN6 or to PSTPIP1. Some of these proteins, such as BTK, SYK or myosin-9 and myosin-I, which bind the BAR domain of PSTPIP1, might interact with the PTPN6 SH2 domains. In B-cells, PTPN6 dephosphorylates BTK and SYK thereby decreasing their kinase activity [32]. We have also identified several proteins belonging to the interactome of the PSTPIP1 SH3 domain, such as the tyrosine-protein kinases ABL1/2, the actin nucleation promoting factors like WASP, VASP, WIPF1 or WASL (N-WASP), the E3 ubiquitin protein ligases Cbl,. PTPNs guided by PSTPIP can dephosphorylate WASP [33].

PSTPIP1 form a complex with PTPN6 and the PI(3,4,5)P3, 5 phosphatases SHIP1/2 which all control sealing zone dynamics. The phosphorylation state of SHIP1 is affected by Phenylarsine oxide, a PTPN inhibitor, or after PSTPIP1 or PTPN6 knockdown. It is likely that a PTPN6-mediated dephosphorylation of SHIP1/2 increases their activity and decrease the levels of PI(3,4,5)P3 on podosomes and sealing zones. This would be consistent with the 10–15 fold increase in PI(3,4,5)P3 levels observed in PTPN6-deficient macrophages [29]. Thus, the PSTPIP1/PTPN6/SHIP1/2 complex could function in podosome dynamic instability by dephosphorylating tyrosines of key podosome components, and by decreasing PI(3,4,5)P3 levels, whose synthesis is linked with integrin engagement [34].

The dynamic instability of sealing zones affects osteoclast function. Our results show that osteoclasts unable to assemble podosomes and sealing zones, as seen after Src [7], PSTPIP2, PTPN12 or PTPN22 depletion, are also unable to digest osteological discs. In contrast, osteoclasts with a reduced ability to disassemble sealing zones, as seen after PSTPIP1, PTPN6, SHIP1 or SHIP2 depletion, exhibit a higher capability to digest such surfaces. This higher capacity in digestion most likely reflects the fact that osteoclasts with reduced sealing zone dynamics remain longer at every given place, which is more efficiently digested. Thus, the dynamic instability of podosomes and sealing zones has a strong impact in osteoclast activity in digestion. In agreement with this, SHIP1 deficient mice are severely osteoporotic due to increased numbers of hyper-resorptive osteoclasts [35]. Mutations that abrogate PSTPIP2 expression in *Lupo* mice lead to auto-inflammatory disease involving extra-medullary hematopoiesis, skin and bone lesions [36]. In addition, osteoclast precursors purified from these mice exhibit increased osteoclastogenesis. This finding would contradict our prediction that PSTPIP2 deficient mice would exhibit osteopetrosis due a lack of function of osteoclasts in degradation. However, Lupo mice have been created by random chemical mutation [12]. Therefore, conditional knockouts in mouse osteoclasts would help understanding the precise function of PSTPIP2 in bone physiology. We generated mice with a conditionally deleted allele of PSTPIP1 induced by tamoxifen treatment. PSTPIP1-/- primary osteoclasts exhibit a higher resorption activity than control osteoclasts.

In conclusion, we illustrate the functional importance of PSTPIP1/2 in the structural organization of podosomes and sealing zone dynamics in osteoclasts. While providing a wealth of information about PSTPIP1/2 interactors, our studies illustrate the importance of a protein complex comprising PSTPIP1, PTPN6 and SHIP1/2 that links changes in membrane shape and the dynamics of F-actin-rich structures, with a negative feedback mechanism controlling Src and PI(3,4,5)P3 signaling to regulate osteoclast activity. These findings contribute to understanding determinant cell biological aspects of bone physiology that have been elusive so far.

## Supporting Information

S1 Fig F-BAR domain containing PSTPIP1/2 induce membrane tubulation. **A** Recombinant PSTPIP1 was incubated with giant unilamellar vesicles as indicated in materials and methods and observed by microscopy. Scale bars, 20 μm. **B** GFP-PSTPIP1 or GFP-PSTPIP2 or GFP were expressed in HEK cells and observed by confocal microscopy as indicated in materials and methods. Scale bars, 50 μm.
(TIF)

S2 Fig Localization of mRFP-PSTPIP2 in osteoclasts. Osteoclasts were grown on osteological discs and transfected with a construct to express mRFP-PSTPIP2 (green). Osteoclasts were then fixed and stained for phalloidin (red) and DAPI (blue). Images were analyzed using the Fiji software. Fluorescence intensities across the indicated white lanes as indicated were plotted (scale bar: 20 μm).
(TIF)

S3 Fig *In vivo* PSTPIP1/2 interactors. Osteoclasts were lysed with detergents as indicated in material and methods and the lysates (≈2mg of proteins) were incubated with anti PSTPIP1 or anti PSTPIP2 antibodies and then ProteinA-beads. The immunoprecipitates were analyzed by western blotting using the indicated antibodies. The figures presented are representative of at least 3 independent experiments.
(TIF)

S4 Fig PTPN12 and PTPN22 localization and effect of their depletion on sealing zone dynamics. **A** Non treated osteoclasts or osteoclasts treated with siRNAs targeting PSTPIP1 were grown on osteological discs, then fixed and stained with anti PTPN12 or anti PTPN22 antibodies (green) and phalloidin (red) (scale bars: 50μm). Images were analyzed using the Fiji software. Fluorescence intensities across the indicated white lanes are indicated and Pearson’s coefficients were calculated (0.56 for PTPN22, 0.49 for PTPN12). **B, C** Sealing zone dynamics in PTPN12-or PTPN22-depleted osteoclasts. Osteoclasts were treated with siRNAs targeting PTPN12 or PTPN22 and then plated on osteological discs. After 24 hours, they were infected with a recombinant adenovirus encoding the mRFP-Ezrin actin-binding domain. After 32 hours, osteoclasts were observed by time-lapse videomicroscopy (100 msec. per frame, 1 frame per 1 min., see S8–S10 Movies). The knockdown efficacies were determined and quantified by western blotting. The figures presented are representative of at least 3 independent experiments (mean ± SD).
(TIF)

S5 Fig Effect of PSTPIP1 and PTPN6 depletion on SHIP1 and SHIP2 tyrosine phosphorylation. Phosphotyrosine specific immunoprecipitation of osteoclast lysates from control and PSTPIP1 or PTPN6 knockdown was done and analyzed by Western blot. Magnitude of these changes was spectrometrically measured in 3 independent experiments. Statistical significance of relative values was tested using students t-test. (mean ± SD, * p< 0.05; ** p<0.01).
(TIF)

S1 Movie Dynamics of sealing zones visualized with the mRFP-ezrin actin binding domain in control osteoclasts. (AVI)

S2 Movie Dynamics of sealing zones visualized with the mRFP-ezrin actin binding domain in PSTPIP1-depleted osteoclasts. (AVI)

S3 Movie Dynamics of PI(3, 4,5)P3 and actin at podosomes visualized with the GFP-PI3,4,5 P3 binding domain of Akt and with the mRFP-ezrin actin binding domain in osteoclasts grown on glass coverslips. (AVI)

S4 Movie Dynamics of PSTPIP1 at podosomes visualized with the GFP-PSTPIP1 and with the mRFP-ezrin actin binding domain in osteoclasts grown on glass coverslips. (AVI)

S5 Movie Dynamics of GFP-PSTPIP1 and mRFP-PSPTIP2 at podosomes of osteoclasts grown on glass coverslips. (AVI)

S6 Movie Dynamics of sealing zones visualized with the mRFP-ezrin actin binding domain in control osteoclasts. (AVI)

S7 Movie Dynamics of sealing zones visualized with the mRFP-ezrin actin binding domain in PTPN6-depleted osteoclasts. (AVI)

S8 Movie Dynamics of sealing zones visualized with the mRFP-ezrin actin binding domain in control osteoclasts. (AVI)

S9 Movie Dynamics of sealing zones visualized with the mRFP-ezrin actin binding domain in PTPN12-depleted osteoclasts. (AVI)

S10 Movie Dynamics of sealing zones visualized with the mRFP-ezrin actin binding domain in PTPN22-depleted osteoclasts. (AVI)

S11 Movie Dynamics of sealing zones visualized with the mRFP-ezrin actin binding domain in control osteoclasts. (AVI)

S12 Movie Dynamics of sealing zones visualized with the mRFP-ezrin actin binding domain in SHIP1-depleted osteoclasts. (AVI)

S13 Movie Dynamics of sealing zones visualized with the mRFP-ezrin actin binding domain in SHIP2-depleted osteoclasts. (AVI)

S1 Table PSTPIP2 interactors. Recombinant GST-PSTPIP2 was incubated with osteoclast lysates. Bound proteins were isolated on Glutathione beads, resolved by SDS-PAGE and identified by semi-quantitative mass spectrometry analysis based on MS2 spectral counting. Protein names, gene names, and accession numbers are indicated. MS2 spectral counts are represented in the last column.
(DOC)

## References

[1] Teitelbaum SL. Bone resorption by osteoclasts. Science. 2000;289(5484):1504–8. Epub 2000/09/01. 8789 [pii]. . 10968780 10.1126/science.289.5484.1504

[2] Teitelbaum SL. The osteoclast and its unique cytoskeleton. Ann N Y Acad Sci. 2011;1240:14–7. Epub 2011/12/17. 10.1111/j.1749-6632.2011.06283.x . 22172034

[3] Saltel F, Destaing O, Bard F, Eichert D, Jurdic P. Apatite-mediated actin dynamics in resorbing osteoclasts. Mol Biol Cell. 2004;15(12):5231–41. Epub 2004/09/17. 10.1091/mbc.E04-06-0522 E04-06-0522 [pii]. 15371537 PMC532006

[4] Cox S, Rosten E, Monypenny J, Jovanovic-Talisman T, Burnette DT, Lippincott-Schwartz J, et al Bayesian localization microscopy reveals nanoscale podosome dynamics. Nat Methods. 2012;9(2):195–200. Epub 2011/12/06. nmeth.1812 [pii] 10.1038/nmeth.1812 22138825 PMC3272474

[5] Luxenburg C, Addadi L, Geiger B. The molecular dynamics of osteoclast adhesions. Eur J Cell Biol. 2006;85(3–4):203–11. Epub 2005/12/20. S0171-9335(05)00195-0 [pii] 10.1016/j.ejcb.2005.11.002 . 16360241

[6] Murphy DA, Courtneidge SA. The 'ins' and 'outs' of podosomes and invadopodia: characteristics, formation and function. Nat Rev Mol Cell Biol. 2011;12(7):413–26. Epub 2011/06/24. nrm3141 [pii] 10.1038/nrm3141 21697900 PMC3423958

[7] Lowell CA, Soriano P. Knockouts of Src-family kinases: stiff bones, wimpy T cells, and bad memories. Genes Dev. 1996;10(15):1845–57. Epub 1996/08/01. . 8756343 10.1101/gad.10.15.1845

[8] Heckel T, Czupalla C, Expirto Santo AI, Anitei M, Arantzazu Sanchez-Fernandez M, Mosch K, et al Src-dependent repression of ARF6 is required to maintain podosome-rich sealing zones in bone-digesting osteoclasts. Proc Natl Acad Sci U S A. 2009;106(5):1451–6. Epub 2009/01/24. 0804464106 [pii] 10.1073/pnas.0804464106 19164586 PMC2635769

[9] Chitu V, Stanley ER. Pombe Cdc15 homology (PCH) proteins: coordinators of membrane-cytoskeletal interactions. Trends Cell Biol. 2007;17(3):145–56. 10.1016/j.tcb.2007.01.003 . 17296299

[10] McMahon HT, Gallop JL. Membrane curvature and mechanisms of dynamic cell membrane remodelling. Nature. 2005;438(7068):590–6. Epub 2005/12/02. nature04396 [pii] 10.1038/nature04396 . 16319878

[11] Futterer K, Machesky LM. "Wunder" F-BAR domains: going from pits to vesicles. Cell. 2007;129(4):655–7. Epub 2007/05/22. S0092-8674(07)00597-1 [pii] 10.1016/j.cell.2007.05.006 . 17512400

[12] Grosse J, Chitu V, Marquardt A, Hanke P, Schmittwolf C, Zeitlmann L, et al Mutation of mouse Mayp/Pstpip2 causes a macrophage autoinflammatory disease. Blood. 2006;107(8):3350–8. 10.1182/blood-2005-09-3556 16397132 PMC1895761

[13] Ferguson PJ, Bing X, Vasef MA, Ochoa LA, Mahgoub A, Waldschmidt TJ, et al A missense mutation in pstpip2 is associated with the murine autoinflammatory disorder chronic multifocal osteomyelitis. Bone. 2006;38(1):41–7. Epub 2005/08/27. S8756-3282(05)00278-4 [pii] 10.1016/j.bone.2005.07.009 16122996 PMC3726202

[14] He TC, Zhou S, da Costa LT, Yu J, Kinzler KW, Vogelstein B. A simplified system for generating recombinant adenoviruses. Proc Natl Acad Sci U S A. 1998;95(5):2509–14. 9482916 10.1073/pnas.95.5.2509 PMC19394

[15] Starnes TW, Bennin DA, Bing X, Eickhoff JC, Grahf DC, Bellak JM, et al The F-BAR protein PSTPIP1 controls extracellular matrix degradation and filopodia formation in macrophages. Blood. 2014;123(17):2703–14. 10.1182/blood-2013-07-516948 24421327 PMC3999755

[16] Schmidt A, Rutledge SJ, Endo N, Opas EE, Tanaka H, Wesolowski G, et al Protein-tyrosine phosphatase activity regulates osteoclast formation and function: inhibition by alendronate. Proc Natl Acad Sci U S A. 1996;93(7):3068–73. 8610169 10.1073/pnas.93.7.3068 PMC39762

[17] Sanchez-Fernandez MA, Sbacchi S, Correa-Tapia M, Naumann R, Klemm J, Chambon P, et al Transgenic mice for a tamoxifen-induced, conditional expression of the Cre recombinase in osteoclasts. PLoS One. 2012;7(5):e37592 Epub 2012/05/25. 10.1371/journal.pone.0037592 PONE-D-12-03993 [pii]. 22624050 PMC3356310

[18] Daumke O, Roux A, Haucke V. BAR Domain Scaffolds in Dynamin-Mediated Membrane Fission. Cell. 2014;156(5):882–92. Epub 2014/03/04. S0092-8674(14)00211-6 [pii] 10.1016/j.cell.2014.02.017 . 24581490

[19] Mim C, Unger VM. Membrane curvature and its generation by BAR proteins. Trends Biochem Sci. 2012;37(12):526–33. Epub 2012/10/13. S0968-0004(12)00138-7 [pii] 10.1016/j.tibs.2012.09.001 23058040 PMC3508348

[20] Tarone G, Cirillo D, Giancotti FG, Comoglio PM, Marchisio PC. Rous sarcoma virus-transformed fibroblasts adhere primarily at discrete protrusions of the ventral membrane called podosomes. Exp Cell Res. 1985;159(1):141–57. Epub 1985/07/01. S0014-4827(85)80044-6 [pii]. . 2411576 10.1016/s0014-4827(85)80044-6

[21] Linder S, Kopp P. Podosomes at a glance. J Cell Sci. 2005;118(Pt 10):2079–82. Epub 2005/05/14. 118/10/2079 [pii] 10.1242/jcs.02390 . 15890982

[22] Ochoa GC, Slepnev VI, Neff L, Ringstad N, Takei K, Daniell L, et al A functional link between dynamin and the actin cytoskeleton at podosomes. J Cell Biol. 2000;150(2):377–89. Epub 2000/07/26. 10908579 10.1083/jcb.150.2.377 PMC2180219

[23] Nitsch L, Gionti E, Cancedda R, Marchisio PC. The podosomes of Rous sarcoma virus transformed chondrocytes show a peculiar ultrastructural organization. Cell Biol Int Rep. 1989;13(11):919–26. Epub 1989/11/01. . 2557979 10.1016/0309-1651(89)90074-x

[24] Bruzzaniti A, Neff L, Sanjay A, Horne WC, De Camilli P, Baron R. Dynamin forms a Src kinase-sensitive complex with Cbl and regulates podosomes and osteoclast activity. Mol Biol Cell. 2005;16(7):3301–13. Epub 2005/05/06. E04-12-1117 [pii] 10.1091/mbc.E04-12-1117 15872089 PMC1165412

[25] Tsujita K, Kondo A, Kurisu S, Hasegawa J, Itoh T, Takenawa T. Antagonistic regulation of F-BAR protein assemblies controls actin polymerization during podosome formation. J Cell Sci. 2013;126(Pt 10):2267–78. 10.1242/jcs.122515 . 23525018

[26] Zou W, Izawa T, Zhu T, Chappel J, Otero K, Monkley SJ, et al Talin1 and Rap1 are critical for osteoclast function. Mol Cell Biol. 2013;33(4):830–44. Epub 2012/12/12. MCB.00790-12 [pii] 10.1128/MCB.00790-12 23230271 PMC3571341

[27] Steenblock C, Heckel T, Czupalla C, Espirito Santo AI, Niehage C, Sztacho M, et al The Cdc42 guanine nucleotide exchange factor FGD6 coordinates cell polarity and endosomal membrane recycling in osteoclasts. J Biol Chem. 2014;289(26):18347–59. 10.1074/jbc.M113.504894 24821726 PMC4140270

[28] Vives V, Laurin M, Cres G, Larrousse P, Morichaud Z, Noel D, et al The Rac1 exchange factor Dock5 is essential for bone resorption by osteoclasts. J Bone Miner Res. 2011;26(5):1099–110. Epub 2011/05/05. 10.1002/jbmr.282 . 21542010 PMC4640905

[29] Roach TI, Slater SE, White LS, Zhang X, Majerus PW, Brown EJ, et al The protein tyrosine phosphatase SHP-1 regulates integrin-mediated adhesion of macrophages. Curr Biol. 1998;8(18):1035–8. Epub 1998/09/19. S0960-9822(07)00426-5 [pii]. . 9740804 10.1016/s0960-9822(07)00426-5

[30] Kruger J, Butler JR, Cherapanov V, Dong Q, Ginzberg H, Govindarajan A, et al Deficiency of Src homology 2-containing phosphatase 1 results in abnormalities in murine neutrophil function: studies in motheaten mice. J Immunol. 2000;165(10):5847–59. Epub 2000/11/09. . 11067945 10.4049/jimmunol.165.10.5847

[31] Poole AW, Jones ML. A SHPing tale: perspectives on the regulation of SHP-1 and SHP-2 tyrosine phosphatases by the C-terminal tail. Cell Signal. 2005;17(11):1323–32. Epub 2005/08/09. S0898-6568(05)00123-3 [pii] 10.1016/j.cellsig.2005.05.016 . 16084691

[32] Maeda A, Scharenberg AM, Tsukada S, Bolen JB, Kinet JP, Kurosaki T. Paired immunoglobulin-like receptor B (PIR-B) inhibits BCR-induced activation of Syk and Btk by SHP-1. Oncogene. 1999;18(14):2291–7. Epub 1999/05/18. 10.1038/sj.onc.1202552 . 10327049

[33] Cote JF, Chung PL, Theberge JF, Halle M, Spencer S, Lasky LA, et al PSTPIP is a substrate of PTP-PEST and serves as a scaffold guiding PTP-PEST toward a specific dephosphorylation of WASP. J Biol Chem. 2002;277(4):2973–86. Epub 2001/11/17. 10.1074/jbc.M106428200 M106428200 [pii]. . 11711533

[34] Yu CH, Rafiq NB, Krishnasamy A, Hartman KL, Jones GE, Bershadsky AD, et al Integrin-matrix clusters form podosome-like adhesions in the absence of traction forces. Cell reports. 2013;5(5):1456–68. 10.1016/j.celrep.2013.10.040 24290759 PMC3898747

[35] Takeshita S, Namba N, Zhao JJ, Jiang Y, Genant HK, Silva MJ, et al SHIP-deficient mice are severely osteoporotic due to increased numbers of hyper-resorptive osteoclasts. Nat Med. 2002;8(9):943–9. Epub 2002/08/06. 10.1038/nm752 nm752 [pii]. . 12161749

[36] Chitu V, Nacu V, Charles JF, Henne WM, McMahon HT, Nandi S, et al PSTPIP2 deficiency in mice causes osteopenia and increased differentiation of multipotent myeloid precursors into osteoclasts. Blood. 2012;120(15):3126–35. Epub 2012/08/28. blood-2012-04-425595 [pii] 10.1182/blood-2012-04-425595 22923495 PMC3471520

