# Supplementary figures and images for: BAR Proteins PSTPIP1/2 Regulate Podosome Dynamics and the Resorption Activity of Osteoclasts
Source: PLoS One. 2016 Oct 19;11(10):e0164829. doi: 10.1371/journal.pone.0164829 (PMC5070766; doi:10.1371/journal.pone.0164829)

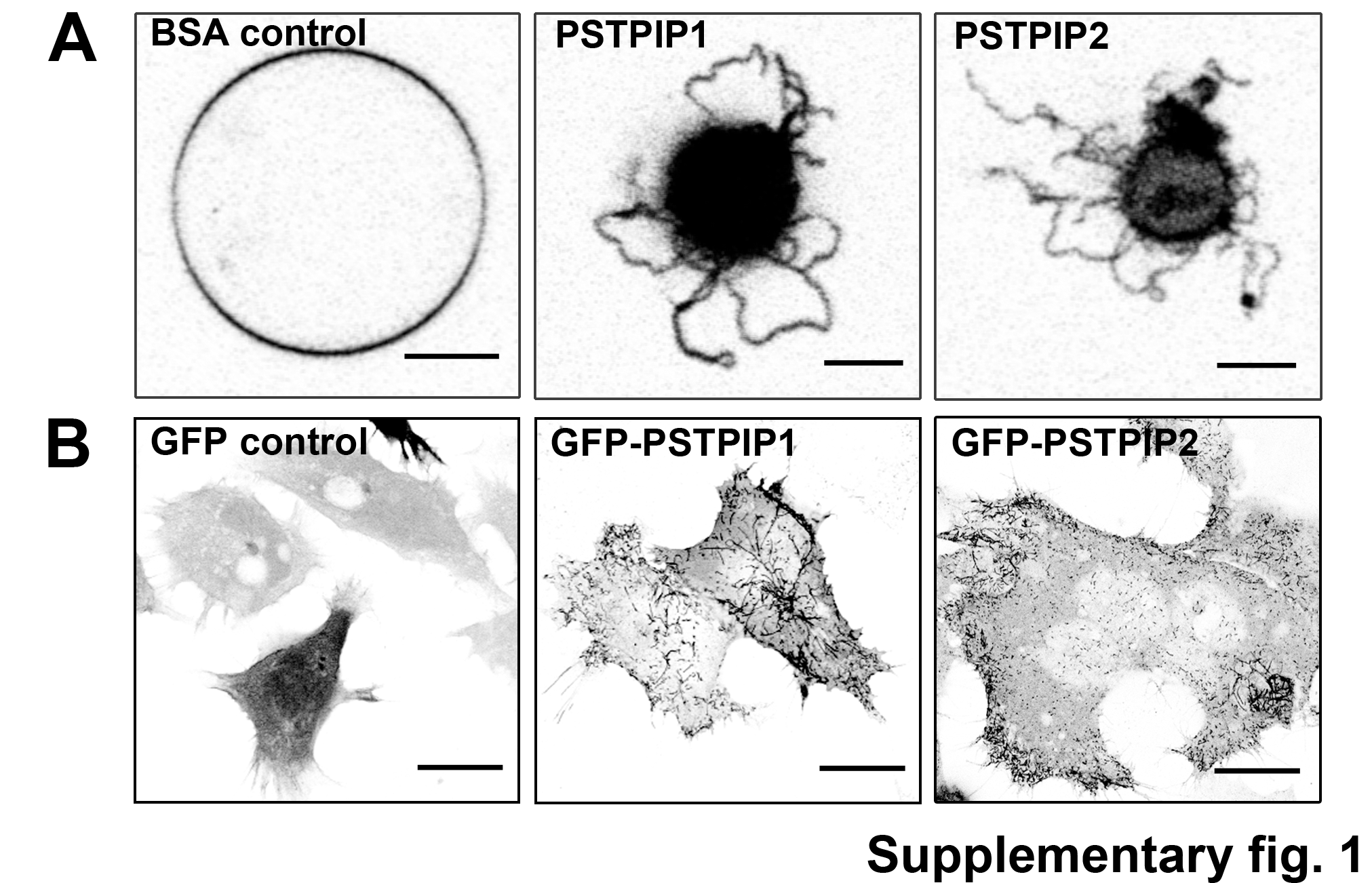

Supplement: S1 Fig — A Recombinant PSTPIP1 was incubated with giant unilamellar vesicles as indicated in materials and methods and observed by microscopy. Scale bars, 20 μm. B GFP-PSTPIP1 or GFP-PSTPIP2 or GFP were expressed in HEK cells and observed by confocal microscopy as indicated in materials and methods. Scale bars, 50 μm. (TIF) [file pone.0164829.s001.tif]

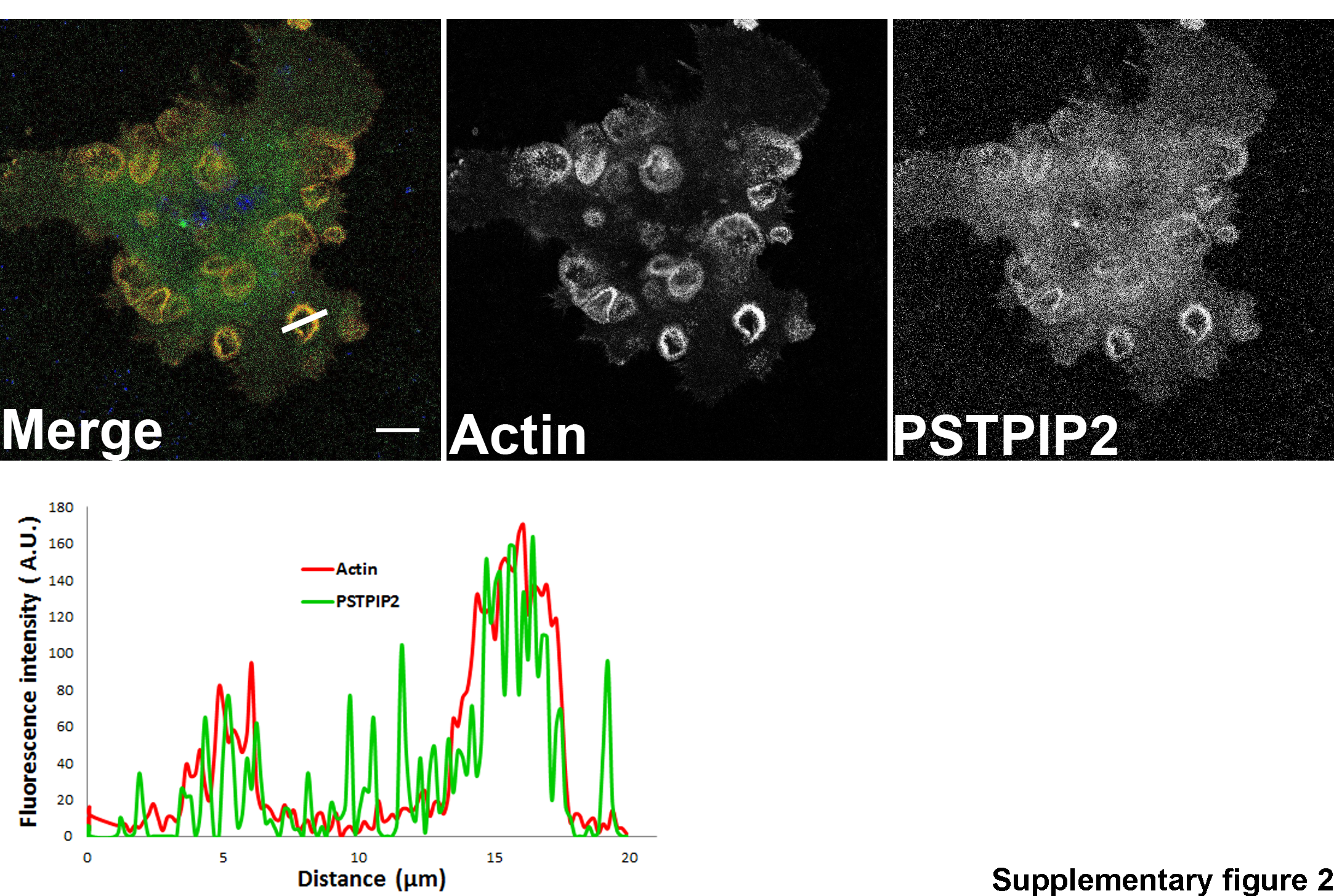

Supplement: S2 Fig — Osteoclasts were grown on osteological discs and transfected with a construct to express mRFP-PSTPIP2 (green). Osteoclasts were then fixed and stained for phalloidin (red) and DAPI (blue). Images were analyzed using the Fiji software. Fluorescence intensities across the indicated white lanes as indicated were plotted (scale bar: 20 μm). (TIF) [file pone.0164829.s002.tif]

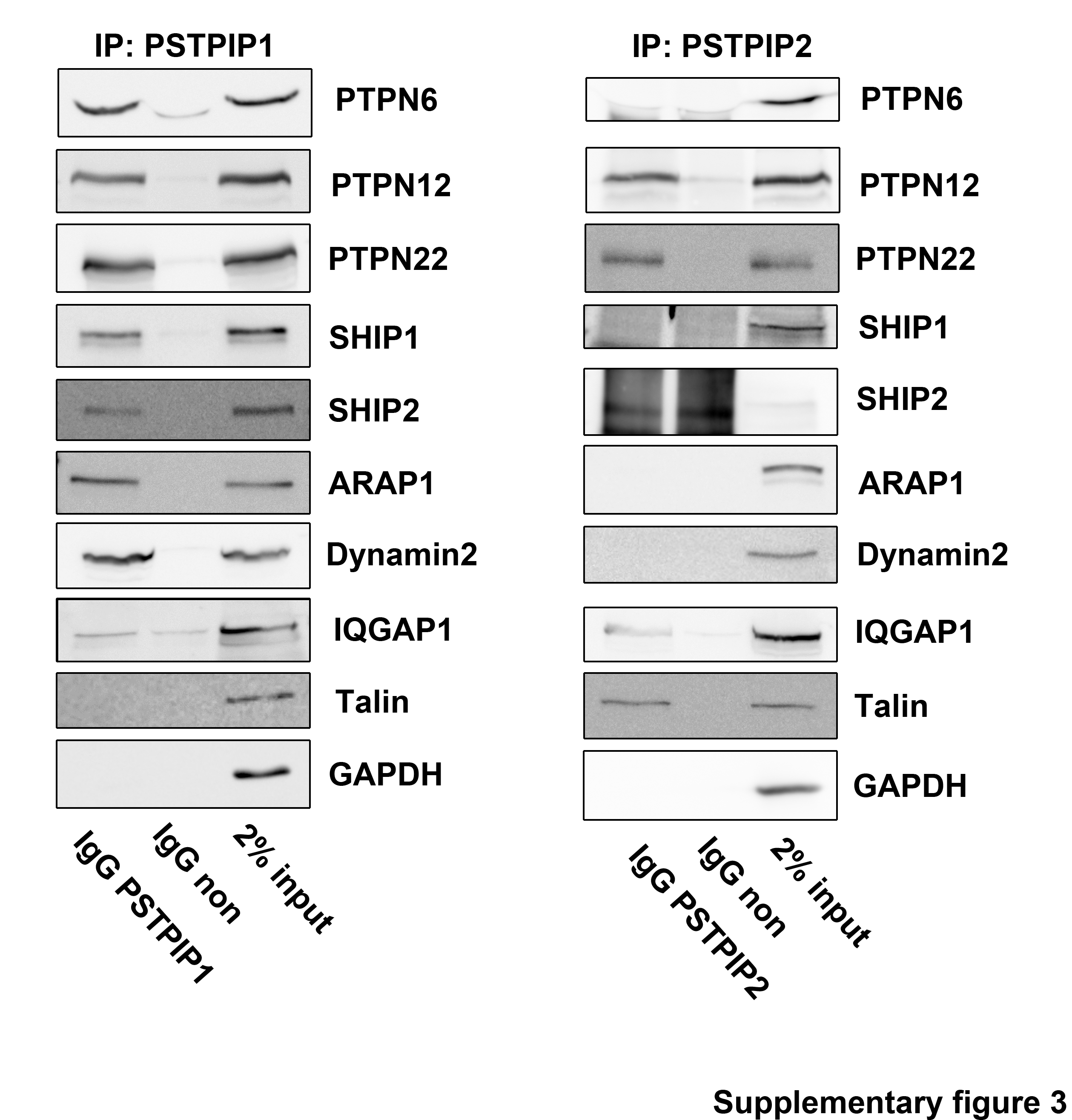

Supplement: S3 Fig — Osteoclasts were lysed with detergents as indicated in material and methods and the lysates (≈2mg of proteins) were incubated with anti PSTPIP1 or anti PSTPIP2 antibodies and then ProteinA-beads. The immunoprecipitates were analyzed by western blotting using the indicated antibodies. The figures presented are representative of at least 3 independent experiments. (TIF) [file pone.0164829.s003.tif]

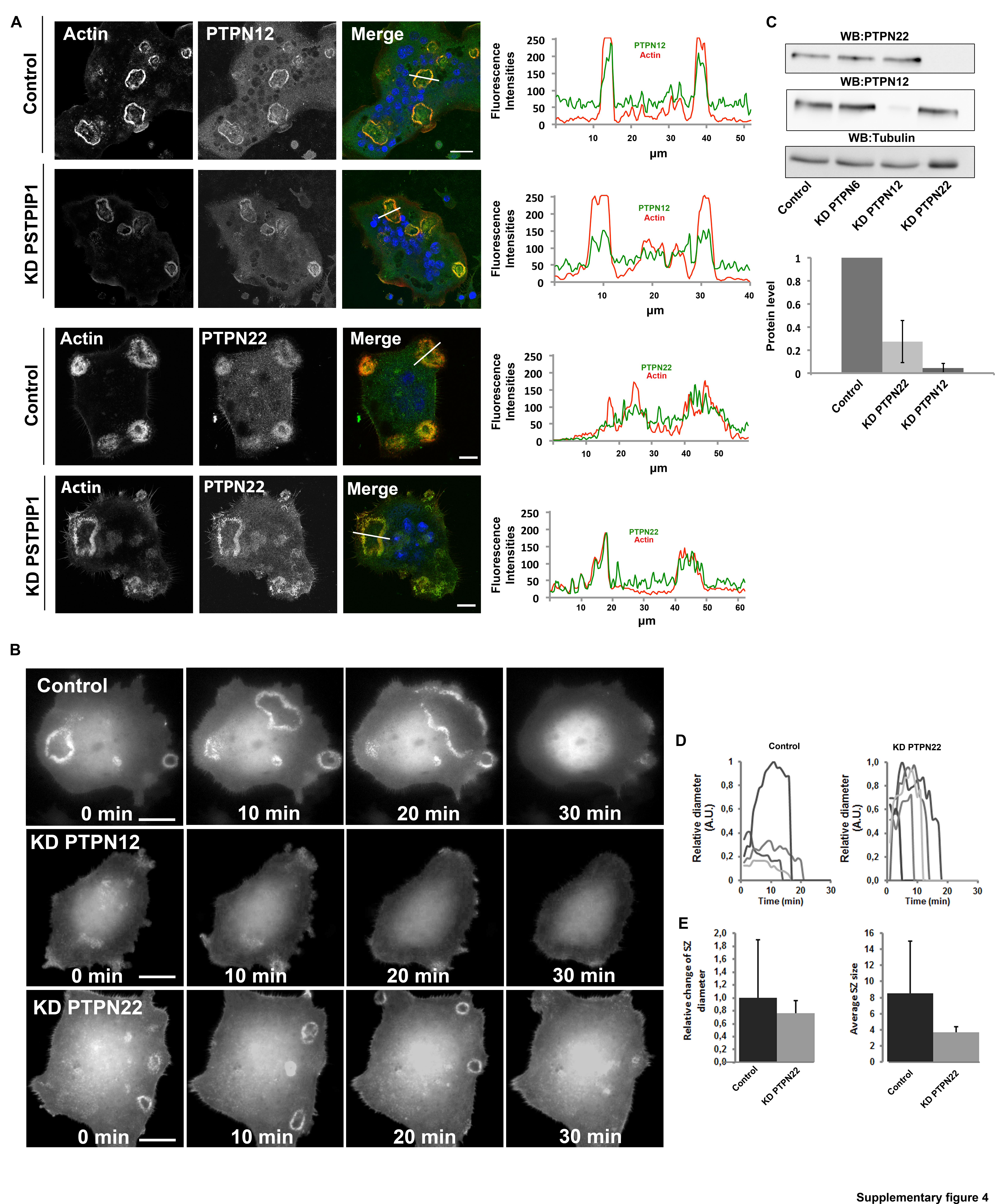

Supplement: S4 Fig — A Non treated osteoclasts or osteoclasts treated with siRNAs targeting PSTPIP1 were grown on osteological discs, then fixed and stained with anti PTPN12 or anti PTPN22 antibodies (green) and phalloidin (red) (scale bars: 50μm). Images were analyzed using the Fiji software. Fluorescence intensities across the indicated white lanes are indicated and Pearson’s coefficients were calculated (0.56 for PTPN22, 0.49 for PTPN12). B, C Sealing zone dynamics in PTPN12-or PTPN22-depleted osteoclasts. Osteoclasts were treated with siRNAs targeting PTPN12 or PTPN22 and then plated on osteological discs. After 24 hours, they were infected with a recombinant adenovirus encoding the mRFP-Ezrin actin-binding domain. After 32 hours, osteoclasts were observed by time-lapse videomicroscopy (100 msec. per frame, 1 frame per 1 min., see S8–S10 Movies). The knockdown efficacies were determined and quantified by western blotting. The figures presented are representative of at least 3 independent experiments (mean ± SD). (TIF) [file pone.0164829.s004.tif]

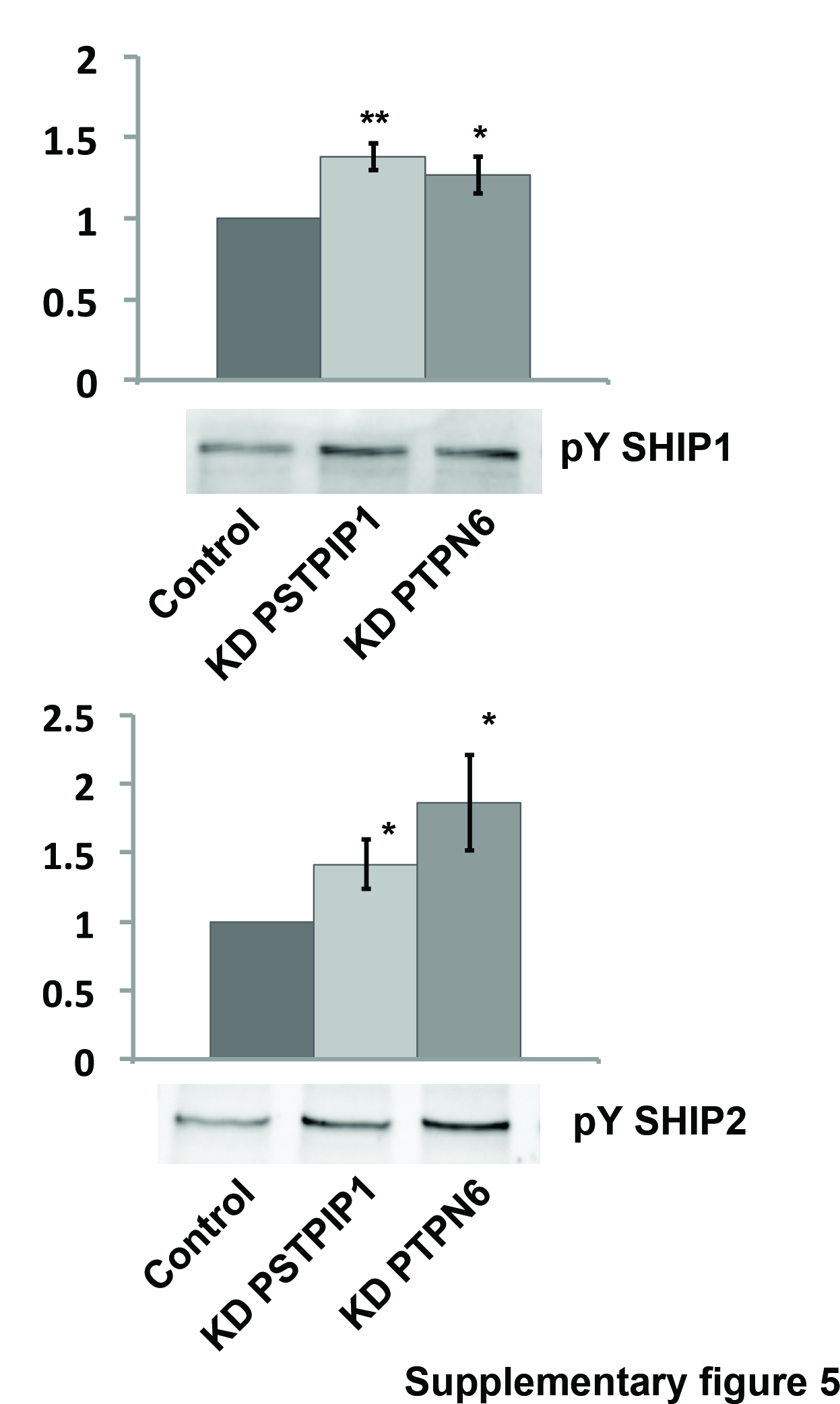

Supplement: S5 Fig — Phosphotyrosine specific immunoprecipitation of osteoclast lysates from control and PSTPIP1 or PTPN6 knockdown was done and analyzed by Western blot. Magnitude of these changes was spectrometrically measured in 3 independent experiments. Statistical significance of relative values was tested using students t-test. (mean ± SD, * p< 0.05; ** p<0.01). (TIF) [file pone.0164829.s005.tif]
