# Supplementary material for: BAR Proteins PSTPIP1/2 Regulate Podosome Dynamics and the Resorption Activity of Osteoclasts
Source: PLoS One. 2016 Oct 19;11(10):e0164829. doi: 10.1371/journal.pone.0164829 (PMC5070766; doi:10.1371/journal.pone.0164829)
Supplement: S1 Table — Recombinant GST-PSTPIP2 was incubated with osteoclast lysates. Bound proteins were isolated on Glutathione beads, resolved by SDS-PAGE and identified by semi-quantitative mass spectrometry analysis based on MS2 spectral counting. Protein names, gene names, and accession numbers are indicated. MS2 spectral counts are represented in the last column. (DOC) [file pone.0164829.s019.doc]

**S1 Table. PSTPIP2 interactors.**

| **Protein name** | **Gene name** | **Acc. number** | **Counts** |
| --- | --- | --- | --- |
| *Phosphatases* |  |  |  |
| Tyrosine-protein phosphatase non-receptor type 12 | PTN12 | P35831 | 117 |
| Tyrosine-protein phosphatase non-receptor type 22 | PTN22 | P29352 | 88 |
| *Kinases* |  |  |  |
| Serine/threonine-protein kinase TAO3 | TAOK3 | Q8BYC6 | 119 |
| Tyrosine-protein kinase BTK | BTK | P35991 | 39 |
| *GTPases, GEFs, GAPs* |  |  |  |
| Arf-GAP with coiled-coi | ACAP2 | Q6ZQK5 | 12 |
| Cell division control protein 42 homolog | CDC42 | P60766 | 11 |
| Dedicator of cytokinesis protein 5 | DOCK5 | B2RY04 | 24 |
| Ras GTPase-activating-like protein IQGAP1 | IQGA1 | Q9JKF1 | 194 |
| Rho guanine nucleotide exchange factor 2 | ARHG2 | Q60875 | 22 |
| *PIPs phosphatases* |  |  |  |
| PI(4,5)P2 phosphodiesterase beta-3 | PLCB3 | P51432 | 11 |
| *Cytoskeleton* |  |  |  |
| Coronin-1B | COR1B | Q9WUM3 | 27 |
| Cytoplasmic dynein 1 heavy chain 1 | DYHC1 | Q9JHU4 | 186 |
| Cytoplasmic FMR1-interacting protein 1 | CYFP1 | Q7TMB8 | 50 |
| Kinectin | KTN1 | Q61595 | 23 |
| Microtubule-actin cross-linking factor 1 | MACF1 | Q9QXZ0 | 29 |
| Myosin-Ic | MYO1C | Q9WTI7 | 19 |
| Myosin-Id | MYO1D | Q5SYD0 | 41 |
| PSTPIP1 | PPIP1 | P97814 | 36 |
| Talin-1 | TLN1 | P26039 | 254 |
